# The Host–Symbiont–Pathogen Triad in *Bathymodiolus azoricus*: The Multifunctional Gill at the Deep-Sea Interface

**DOI:** 10.3390/md24090312

**Published:** 2026-09-06

**Authors:** Raul Bettencourt

**Affiliations:** 1 Faculty of Sciences and Technology, University of the Azores, 9901-862 Horta, Portugal; raul.s.bettencourt@uac.pt; 2 OKEANOS Institute of Marine Sciences, University of the Azores, 9901-862 Horta, Portugal

**Keywords:** *Bathymodiolus azoricus*, innate immunity, holobiont, hydrothermal vent, symbiosis, host–symbiont–pathogen triad, pattern-recognition receptors, gill zonation, hemocyte, deep-sea

## Abstract

Deep-sea hydrothermal vents and cold seeps sustain highly productive animal communities through chemosynthetic symbioses, among which bathymodioline mussels are prominent examples. Bathymodioline gill bacteriocytes accommodate intracellular chemosynthetic symbionts, including sulfur- and/or methane-oxidizing bacteria depending on the host species, while remaining sheltered in an epithelium continuously exposed to environmental microorganisms, creating a fundamental immunological problem: how can an innate defense system remain effective without eliminating the microbial partners on which host nutrition depends? This review examines this problem through *Bathymodiolus azoricus*, integrating two decades of work on its cellular immunity, gill transcriptome, microbial challenge responses and symbiosis biology with recent mechanistic studies from related bathymodiolines. Central to the present synthesis are previously reported *B. azoricus* observations showing that gill tissue can mount local transcriptional responses to bacterial challenge, while hemolymph serum differentially modulates immune-gene expression following exposure to symbiont preparations or non-symbiotic Vibrio. Immune-gene expression also varies along the anterior–posterior gill axis, with lower expression in the posterior budding zone than in mature anterior filaments. We interpret this zonation primarily as a feature of tissue maturation rather than demonstrated active immune suppression, consistent with evidence that newly formed filaments are initially aposymbiotic and become colonized only after formation. Together, these observations evoke a host–symbiont–pathogen triad in which local gill-tissue responses, systemic humoral modulation and gill development constitute interacting levels of immune organization and compartmentalization. As a working hypothesis, we propose that this triad is reconciled principally through spatial and developmental compartmentalization of immune competence rather than through generalized immune suppression, predicting that immune-gene expression should track gill maturation state rather than symbiont occupancy per se. We consider this tissue-level model alongside comparative evidence for putative symbiont-uptake mechanisms, post-engulfment microbial discrimination, lysosomal regulation, symbiont digestion and bacteriocyte turnover, including the mTORC1-dependent phagosome-digestion checkpoint demonstrated in *Bathymodiolus japonicus*. Rather than assuming that these mechanisms are conserved across species, we distinguish explicitly between findings established in *B. azoricus*, evidence from other bathymodiolines and canonical pathways used as mechanistic context. We conclude by identifying unresolved components of *B. azoricus* immunity, including the prophenoloxidase system, the broader antimicrobial-peptide repertoire and the relationship between cellular checkpoints and tissue-level gill zonation, and consider the prospective biotechnological relevance of mechanisms that tolerate persistent microbial symbiosis without loss of immune vigilance.

## 1. Introduction

Nearly five decades have passed since the discovery of hydrothermal-vent ecosystems along the Galápagos Rift revealed animal communities sustained independently of photosynthetic primary production [1]. Subsequent exploration identified high-temperature hydrothermal systems along other spreading centers, including the East Pacific Rise [2]. Vent communities were subsequently documented across diverse geological settings, including mid-ocean ridges and Southwestern Pacific back-arc basins [3,4,5]. These environments are characterized by steep thermal and redox gradients and exposure to reduced compounds, yet they sustain dense animal assemblages through microbial chemosynthesis and chemosynthetic autotrophic symbioses [3,4]. Vent faunas also show marked regional differentiation, with distinct biogeographic provinces and dispersal relationships on a global scale [6]. Chemosynthetic symbioses are not restricted to hydrothermal vents but occur across cold seeps, whale and wood falls, anoxic sediments, and continental margins, and have evolved repeatedly in distantly related animal lineages [7].

Among the best-studied animals from chemosynthetic ecosystems are bathymodioline mussels, whose nutrition can depend substantially on intracellular chemoautotrophic bacteria housed within specialized gill epithelial cells, the bacteriocytes. Symbiont composition varies among lineages: some species associate predominantly with sulfur-oxidizing or methane-oxidizing bacteria [7], whereas *Bathymodiolus azoricus* and *Bathymodiolus puteoserpentis* maintain dual associations with both symbiont types [8]. These relationships have become important models for investigating how environmentally acquired symbionts are recognized, incorporated, maintained, and dynamically regulated under changing physiological conditions. Here, the term holobiont is used in its physiological sense to describe the host together with its associated microbial partners as an interacting biological system, without assuming that host and microbiota necessarily constitute a single unit of evolutionary selection [9,10]. This physiological usage is retained throughout the review; subsequent references to the holobiont perspective or holobiont model describe the interacting host–symbiont system as studied experimentally, not a claim of co-evolutionary integration or joint selection. On the northern Mid-Atlantic Ridge, conspicuous populations of *B. azoricus* thrive at sites such as Menez Gwen and Lucky Strike, where host physiology and symbiont metabolism adapt to highly variable geochemical regimes [8].

Molecular work on *B. azoricus* subsequently established the species as an experimentally manageable model for deep-sea innate immunity and host–microbe interactions. The 2010 gill transcriptome generated one of the earliest large-scale molecular inventories from a deep-sea bivalve and identified numerous transcripts with homology to known innate-immune components [11]. These included candidate pattern-recognition receptors such as Toll-like receptors, PGRPs, C-type lectins, scavenger receptors and SRCR-domain proteins, together with putative intracellular signaling components and transcriptional regulators including MyD88, TRAF6, IRAK-family proteins, NF-κB, AP-1 and STAT. Their presence established that *B. azoricus* possesses a broad molecular repertoire homologous to canonical invertebrate innate-immune systems, while leaving the functional connectivity and symbiosis-specific deployment of many of these components unresolved. Homology-based identification of this kind establishes sequence similarity to known immune components but does not by itself constitute evidence of functional immune activity; functional validation for these candidates in *B. azoricus* remains largely absent.

The relative accessibility of northern Mid-Atlantic Ridge vent fields from the Azores, together with the unusual capacity of *B. azoricus* to remain viable after recovery and decompression, enabled controlled laboratory experimentation that is difficult to support for many deep-sea vent animals. The LabHorta aquarium system allowed live mussels to be maintained at atmospheric pressure for extended periods and provided a reproducible platform for examining post-capture physiology, immune and stress responses, and progressive changes in symbiont abundance under controlled conditions [12,13,14,15,16]. This experimental framework supported ex vivo challenges using dissected gill tissue, bacterial-exposure experiments and long-term acclimatization studies, while necessarily representing a divergence from the pressure and geochemical conditions experienced in situ.

A central paradox running through this body of work is how an innate immune system capable of recognizing and controlling potentially harmful microorganisms can simultaneously accommodate intracellular bacterial partners on which host nutrition depends. Resolving this apparent tension between symbiont maintenance and pathogen defense is the organizing question of this review.

We formalize this tension as a host–symbiont–pathogen triad, in which local gill-tissue sensing, systemic humoral modulation, and gill development interact to determine immune outcome at the symbiotic interface (Section 5). As a working hypothesis, we propose that this triad is reconciled principally through spatial and developmental compartmentalization of immune competence, rather than through generalized suppression of immune reactivity in symbiont-bearing tissue. This hypothesis predicts that immune-gene expression should track filament maturation state rather than symbiont occupancy per se —a prediction directly testable by comparing age-matched aposymbiotic and colonized filament regions in *B. azoricus*, a comparison not yet available.

The gill provides the most direct tissue context in which this problem can be examined. Beyond its respiratory and feeding functions, the gill epithelium houses symbiont-bearing bacteriocytes while remaining continuously exposed to microorganisms carried across the epithelial surface by seawater [11,12]. Nutritional symbiosis and antimicrobial surveillance therefore occur within the same organ and, in part, within the same cellular landscape. For this reason, the present review places particular emphasis on the gill epithelium as the interface at which microbial recognition, intracellular symbiont maintenance and pathogen-responsive immunity converge.

Subsequent high-throughput studies across bathymodioline species broadened this molecular inventory and increasingly shifted attention from the mere presence of immune genes toward the cellular processes governing microbial retention or removal. The specific comparative evidence for these processes is considered in Section 6 and Section 7. Importantly, direct cell-biological work in *B. japonicus* showed that gill epithelial cells can engulf both symbiotic and non-symbiotic bacteria and that discrimination can emerge after uptake through differential phagosome processing [17]. Whether equivalent post-engulfment selectivity operates in *B. azoricus* remains to be demonstrated.

Recent advances have brought the bathymodioline symbiosis into substantially finer cellular resolution. Single-nucleus transcriptomics in *Gigantidas platifrons* (formerly classified within Bathymodiolus; both generic names appear in the cited literature, and this review retains the name used by each source while defaulting to Gigantidas when referring to the species independent of a specific citation) has resolved diverse gill cell populations and developmental states [18], while direct cell-biological studies in *B. japonicus* have identified nutrient-sensitive regulation of symbiont digestion within bacteriocytes [17,19]. In particular, experimental work demonstrated that mTORC1 regulates phagosome digestion in *B. japonicus*, linking symbiont-derived nutritional status to intracellular retention or degradation [19]; this mechanism was subsequently addressed in the 2025 review by Tame [20]. These findings provide a cellular-level framework against which previously reported *B. azoricus* observations can be reconsidered. The present review therefore asks whether the anterior–posterior differences in immune-gene expression described in *B. azoricus* represent a complementary tissue-level layer of organization and how, if at all, such zonation relates to the cellular checkpoints identified in other bathymodioline species.

This review revisits *B. azoricus* as a model for investigating innate immunity within a chemosynthetic symbiosis. We first outline the development of the species as an experimental system (Section 2) and summarize the cellular and molecular immune repertoire identified through physiological and transcriptomic studies (Section 3 and Section 4). We then reassess previously reported ex vivo and spatial-expression observations from *B. azoricus* within a host–symbiont–pathogen framework, with particular emphasis on hemolymph-dependent modulation and anterior–posterior variation in gill immune-gene expression (Section 5). These findings are compared with cellular and molecular mechanisms resolved in other bathymodioline species, including candidate uptake pathways, post-engulfment microbial processing, nutrient-sensitive symbiont digestion and bacteriocyte turnover (Section 6). We subsequently integrate these tissue- and cell-level observations into a multiscale conceptual model (Section 7), consider their prospective biotechnological relevance (Section 8), and identify methodological limitations and unresolved questions that should guide future work (Section 9). Throughout, we adopt an explicit evidence-grading convention: findings demonstrated directly in *B. azoricus* are distinguished from observations inferred from *B. azoricus* transcriptomic or expression data without functional validation, from mechanisms experimentally demonstrated in other bathymodioline species, from canonical pathways retained for mechanistic context only, and from hypotheses that remain to be tested. This distinction is signaled explicitly at the point each finding is introduced, rather than reserved for the Abstract and Conclusions.

## 2. The Emergence of *Bathymodiolus azoricus* as a Model System

Early work on *B. azoricus* established the species as an unusually amenable model for investigating innate immunity and physiological responses in a deep-sea chemosymbiotic animal. Unlike many vent organisms that are difficult to maintain following recovery, *B. azoricus* can remain viable for extended periods after decompression and under atmospheric-pressure aquarium conditions, although this necessarily represents a significant departure from its natural hydrostatic pressure and geochemical environment. This resilience enabled controlled laboratory exposure experiments involving bacteria, environmental stressors and post-capture acclimatization, and created an experimental framework in which immune and stress responses could be examined outside the deep-sea habitat [12,13,14,15,16].

These early studies were followed by large-scale transcriptomic sequencing of *B. azoricus* gill tissue, which generated an extensive catalog of expressed genes and provided a molecular basis for investigating innate immunity in a deep-sea chemosymbiotic bivalve [11]. As detailed in Section 4, this normalized, poly(A)-selected, early-generation sequencing resource is most appropriately regarded as a gene-discovery tool rather than a quantitative or fully validated inventory, and this caveat applies to all transcriptomic findings summarized below. The dataset identified numerous transcripts homologous to components of established invertebrate immune systems, including Toll-like receptors, peptidoglycan-recognition proteins, C-type lectins, scavenger and SRCR-domain receptors, together with putative intracellular signaling molecules such as MyD88, TRAF6 and IRAK-family proteins, transcriptional regulators including NF-κB, AP-1 and STAT, and candidate antimicrobial effectors. These findings demonstrated that *B. azoricus* possesses a broad innate-immune molecular repertoire comparable in overall organization to that described in other bivalves [12]. They did not, however, establish how these components are functionally connected or how they distinguish symbiotic from potentially harmful microorganisms. Those mechanistic questions emerged progressively from subsequent experimental and comparative studies and form the central focus of the present review.

**Figure 1:**
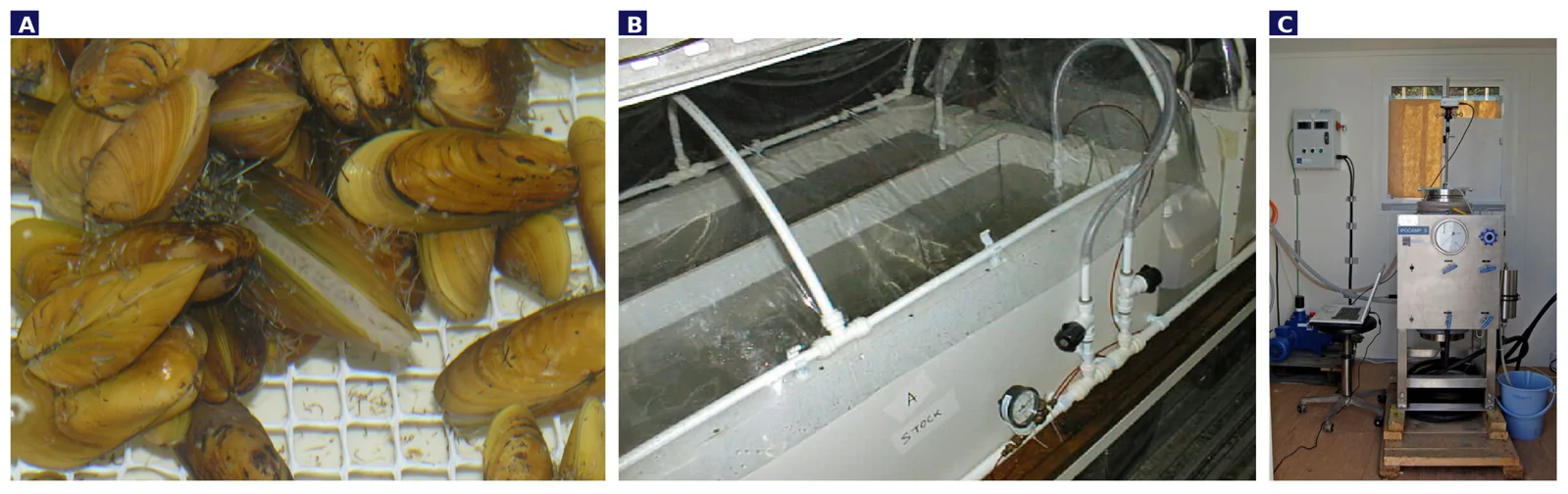
The LabHorta facility (OKEANOS Marine Research Institute, University of the Azores), shown here at its former quarters within the Department of Oceanography and Fisheries, prior to the 2015 inauguration of the OKEANOS Institute, used for long-term maintenance and experimental manipulation of *Bathymodiolus azoricus* under laboratory conditions. (**A**) Live *B. azoricus* specimens recovered from the Menez Gwen hydrothermal vent field, shown shortly after arrival at LabHorta. (**B**) The flow-through aquarium system supplemented with methane and sodium sulfide (Na_2_S) solution, used to maintain mussels at atmospheric pressure for extended periods, permitting controlled experimentation on immune and stress responses under stable laboratory conditions. (**C**) The IPOCAMP pressurization/decompression vessel, used to subject specimens to hydrostatic pressures approximating in situ conditions recorded at the Menez Gwen and Lucky Strike vent fields, allowing pressure-dependent physiological and immune responses to be examined experimentally.

Subsequent work using the LabHorta aquarium system extended these molecular observations into controlled experimental settings [12,15,16]. Live *B. azoricus* could be maintained for prolonged periods at atmospheric pressure, allowing post-capture physiological changes, immune-gene expression, stress responses and changes in gill symbiont abundance to be followed experimentally (Figure 1). These studies provided the foundation for bacterial challenge assays, analyses of hemocyte activity and phagocytosis, measurements of apoptosis-related responses, and investigations of immune- and stress-associated gene expression in vivo and ex vivo [13,14,15]. They also established an important methodological context for later work, i.e., although *B. azoricus* retains measurable immune responsiveness under aquarium conditions, long-term acclimatization is accompanied by physiological and symbiotic changes that must be considered when extrapolating laboratory observations to the natural vent environment [15,16].

Subsequent studies showed that *B. azoricus* retains inducible immune- and stress-associated responses under atmospheric-pressure laboratory conditions, despite the absence of the hydrostatic pressure and reduced compounds characteristic of the vent habitat. Rapid thermal change induces broad transcriptional responses in *B. azoricus*, further illustrating the physiological plasticity of the species [21]. At the same time, metabolic studies reinforced the dependence of the host on its chemosynthetic partners for organic carbon and other metabolites, with sulfur- and methane-oxidizing symbiont activity depending on the availability of their respective reduced substrates and other environmental conditions [8,12,22].

A more mechanistic link between symbiont nutritional status and intracellular processing has emerged from later work in other bathymodioline species. In *Bathymodiolus japonicus*, mTORC1-dependent regulation of phagosome digestion couples symbiont-derived nutritional signals to the decision to retain or digest intracellular bacteria [19,20]. This mechanism provides a valuable comparative framework for interpreting *B. azoricus* observations, but it has not yet been demonstrated directly in this species. The present review therefore treats the *B. japonicus* checkpoint as complementary comparative evidence rather than as an established mechanism of *B. azoricus* symbiosis.

## 3. Cellular Innate Immunity in *B. azoricus*

### 3.1. Hemocytes and Hemolymph-Associated Immunity

Early cellular studies characterized the hemolymph and extrapallial fluid of *B. azoricus* and distinguished three hemocyte types, with granulocytes representing the most abundant class [23]. Fluorescent wheat-germ agglutinin (WGA) binding was detected predominantly on granulocytes, providing an additional marker for hemocyte discrimination alongside morphology. Functional assays further examined mitogen-activated protein kinase (MAPK) activation, intracellular Ca^2+^ dynamics and phagocytosis following exposure to microbial compounds, fluorescent yeast particles and live bacteria [23]. These observations identified granulocytes as a major phagocytic and microbe-responsive hemocyte population in *B. azoricus*. However, the functional specialization of the different hemocyte classes remains incompletely resolved, as does their relationship to soluble hemolymph factors that modulate gill immune responses during microbial challenge. In addition to circulating hemocytes, the cell-free serum fraction of the hemolymph contains soluble components capable of modifying tissue-level immune responses, an effect examined directly in the ex vivo gill experiments discussed in Section 5. The central role of hemocytes in bivalve immune defense is also well established across the class [24].

Comparable functional specialization has been demonstrated in other bathymodioline mussels. In *B. japonicus*, *B. platifrons*, and *B. septemdierum*, three major hemocyte types were distinguished, with agranulocytes dominating the circulating population but showing little or no phagocytic activity, whereas phagosome and phagolysosome formation occurred predominantly in eosinophilic granulocytes [25]. Subsequent monoclonal-antibody profiling in *B. japonicus* further revealed immunophenotypic heterogeneity within both granulocyte and agranulocyte populations, providing molecular markers for future analyses of hemocyte differentiation and localization [26]. These comparative observations support functional heterogeneity among bathymodioline hemocytes, although equivalent cell-type-specific functions remain incompletely resolved in *B. azoricus*.

### 3.2. Pattern-Recognition Receptors and Candidate Signaling Components

The *B. azoricus* gill transcriptome revealed a broad repertoire of transcripts homologous to established components of invertebrate innate immunity [11]. Candidate recognition molecules included Toll-like receptors (TLRs), peptidoglycan-recognition proteins (PGRPs) [27], C-type lectins [28], scavenger receptors and proteins containing scavenger-receptor cysteine-rich (SRCR) domains [29]. Transcripts homologous to intracellular signaling components, including MyD88, TRAF6 and IRAK-family proteins, were also identified, together with transcriptional regulators such as NF-κB, AP-1 and STAT [11]. This inventory indicates that *B. azoricus* possesses molecular components capable of supporting canonical pattern-recognition and downstream signaling processes. This repertoire is broadly consistent with the molecular diversity described in mussel immunity [30]. However, transcript identification alone does not demonstrate that these molecules form the same signaling cascades characterized in model organisms, nor does it establish their specific roles in symbiont recognition or pathogen discrimination. Functional evidence for differential microbial responses is therefore considered separately in Section 5.

### 3.3. Antimicrobial Peptides

In *B. azoricus*, a mytilin-related transcript was detected using cross-species primers designed against conserved regions of previously characterized *Mytilus mytilin* genes [23]. This approach allowed expression of the amplified fragment to be followed during bacterial exposure, including challenge with Vibrio [23]; it was detected by RT-PCR amplification of a partial transcript region rather than isolation, cloning, or complete sequencing of a distinct *B. azoricus* mytilin gene. The evidence therefore supports the presence and regulated expression of a mytilin-like antimicrobial transcript in *B. azoricus*, whereas the structural and functional characterization of mytilins derives principally from shallow-water Mytilus species [31,32,33].

Beyond mytilin, the AMP repertoire of *B. azoricus* remains incompletely characterized. Myticins and defensins are well established in coastal *Mytilus* species [31,32,33], but comparable functional characterization has not, to our knowledge, been reported for *B. azoricus*. A further data point exists at the sequence level, as a *B. azoricus* big-defensin sequence (BDEF_BATAZ; GenBank HM756150) was included in a comparative analysis of molluscan big defensins and showed approximately 41% amino-acid identity to the *Crassostrea gigas* sequences [34]. This supports the presence of a big-defensin homolog in *B. azoricus* but does not constitute functional characterization of the peptide in this species. The broader evolutionary diversification and functional heterogeneity of big defensins in bivalves has been reviewed in detail [35].

A functionally characterized antibacterial effector has, however, been described in *B. azoricus*. Détrée et al. identified six i-type lysozyme paralogs and found that three were upregulated during experimentally induced symbiont loss, while overall lysozyme activity increased as symbionts were depleted [36]. These findings implicate lysozyme-family effectors in symbiont-associated plasticity, while not establishing any single isoform as a direct determinant of symbiont retention.

## 4. Transcriptomic Resources for *Bathymodiolus azoricus* Gill Immunity

### 4.1. The 2010 Gill Transcriptome

The first large-scale molecular resource for *Bathymodiolus azoricus* gill biology was generated in 2010 using 454 GS-FLX sequencing [11]. The original dataset is deposited in the NCBI Sequence Read Archive under SRA accession SRR067874. The study produced approximately 779,000 reads and more than 75,000 assembled contigs, revealing a broad repertoire of transcripts with homology to proteins involved in microbial recognition, intracellular signaling, stress responses, and antimicrobial defense. This dataset provided the molecular foundation for much of the subsequent work on *B. azoricus* innate immunity and host–symbiont interactions. However, the library was normalized and poly(A)-selected and was generated using early-generation sequencing and annotation approaches. It is therefore most appropriately regarded as a gene-discovery resource rather than a quantitative representation of transcript abundance, and individual gene-family assignments remain subject to the uncertainties inherent to de novo transcriptome assembly and sequence annotation. A subsequent analysis of the gill transcriptomic resource specifically examined transcripts attributable to the intracellular endosymbionts and free-living deep-sea bacteria associated with *B. azoricus*, extending the original host-focused transcriptomic resource to the microbial component of the gill system [37].

### 4.2. Independent 2024 Illumina Sequencing of B. azoricus Gill Tissue

A second, independently generated *B. azoricus* gill RNA-sequencing dataset, sequenced in 2024 from material collected at Menez Gwen in August 2013, provides an opportunity to evaluate transcriptomic support for parts of the earlier immune-gene inventory using substantially deeper sequencing and contemporary annotation approaches (see Section 4.3). The dataset was generated using Illumina sequencing from pooled gill tissue collected at the Menez Gwen hydrothermal vent field and is publicly available through NCBI under BioProject PRJNA1508204, BioSample SAMN62218460, and SRA accession SRR40024054. In the analysis considered here, the assembled Trinity dataset comprised 212,318 transcripts and yielded 44,389 predicted proteins following TransDecoder-based coding-sequence prediction and Trinotate annotation.

Because the 2010 and more recent datasets were generated independently and using different sequencing platforms, concordant recovery of particular gene families provides useful independent transcriptomic support for their presence in *B. azoricus* gill tissue, although it does not constitute functional validation.

### 4.3. Cross-Dataset Support for Immune-Associated Gene Families

Comparison of the two transcriptomic resources strengthens several components of the *B. azoricus* immune inventory. Cathepsin-family lysosomal proteases, integrins, fibrinogen-related proteins, allograft inflammatory factor 1 (AIF1), and members of the expanded C1q/TNF-related protein family are independently recovered in both datasets. The C1q/TNF-related sequences should not be interpreted as evidence for a classical vertebrate TNF-α system; rather, they predominantly reflect the diversified C1q/TNF-domain-containing repertoire characteristic of bivalves. Their independent recovery nevertheless strengthens the transcriptomic evidence that these gene families constitute substantial components of the *B. azoricus* gill transcriptome.

The cathepsin repertoire is particularly relevant to the symbiosis–immunity interface developed later in this review. Multiple cathepsin families were recovered from the earlier dataset, and the deeper 2024 assembly identified an expanded set of cathepsin-domain-containing proteins. This provides *B. azoricus*-specific molecular support for a substantial lysosomal protease repertoire, while not by itself establishing the regulatory mechanisms governing symbiont digestion, retention, or phagosome maturation. Cathepsins are emphasized here, rather than other lysosomal proteases, because they are the lysosomal hydrolase family most directly implicated in the mTORC1-dependent phagosome-digestion pathway discussed in Section 6.4 and Section 7.3; comparable functional linkage to symbiont processing has not yet been established for other lysosomal protease families in Bathymodiolus.

Not all earlier annotations were reproduced. Putative sialic-acid-binding immunoglobulin-like lectin (Siglec) sequences identified in the 2010-derived dataset were not recovered in the 2024 transcriptome under the more recent annotation workflow. This discrepancy does not demonstrate that a Siglec-like gene is absent from the *B. azoricus* genome, because transcript detection depends on expression state, sequencing depth, assembly quality, and annotation criteria. It does, however, mean that the earlier assignment should be regarded as provisional rather than established. Such discrepancies illustrate why transcriptomic gene catalogs should be interpreted as evolving molecular resources rather than definitive inventories.

Taken together, the 2010 gill dataset (SRR067874) and the 2024 Illumina gill dataset (BioProject PRJNA1508204; SRR40024054; BioSample SAMN62218460) provide complementary molecular views of *B. azoricus* immunity. The earlier dataset established the breadth of the immune repertoire, whereas the deeper Illumina resource provides independent transcriptomic corroboration for several gene families and highlights others that remain uncertain. Importantly, neither transcriptome establishes how individual components function during symbiont recognition, pathogen challenge, or intracellular microbial processing. Throughout the following sections, we therefore distinguish between transcriptomic evidence for the presence of candidate immune components and experimental evidence demonstrating their functional deployment during host–symbiont–pathogen interactions.

## 5. The Host–Symbiont–Pathogen Triad in *Bathymodiolus azoricus*: Gill Immune Specificity and Zonation

**Figure 2:**
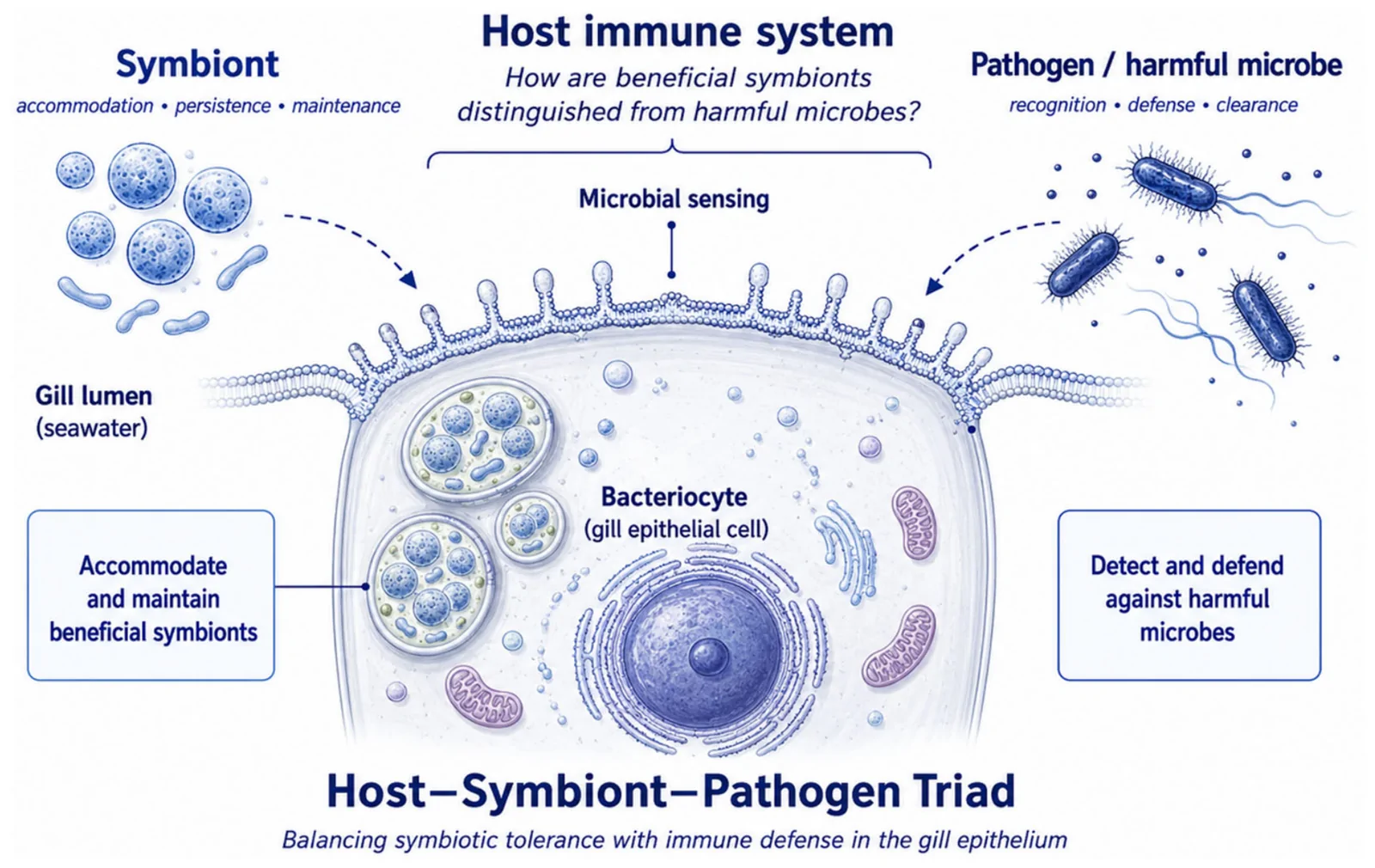
Conceptual framework of the host–symbiont–pathogen triad at the gill interface of bathymodioline mussels. The gill is proposed here as the principal physiological interface at which nutritional symbiosis, immune surveillance, and environmental exposure converge. Within the gill epithelium, bacteriocytes accommodate intracellular chemosynthetic symbionts in symbiont-containing vacuolar compartments, while the apical epithelial surface remains continuously exposed to environmental microorganisms, including potential pathogens [8,11]. This spatial organization creates a fundamental immunological paradox: the host must sustain intracellular symbionts that underpin nutrition and the physiological functioning of the host–symbiont system while remaining competent to recognize and respond to harmful microbes. The triad is therefore presented as a physiological principle integrating microbial recognition, symbiont accommodation and maintenance, and pathogen defense within a single epithelial system. The figure does not depict a demonstrated molecular pathway; rather, it frames the questions addressed throughout this review, including how PRR–MAMP/PAMP signaling and downstream immune modulation may contribute to differential microbial handling, how bacteriocyte trafficking and symbiosome physiology support intracellular persistence and nutrient acquisition, and how symbiont acquisition, maintenance, digestion, loss, and epithelial remodeling are coordinated under changing environmental and nutritional conditions [17,19,20,38,40,41]. Evidence from comparative genomics, transcriptomics, and cell biology is considered in subsequent sections to evaluate how these molecular, cellular, and ecological processes collectively enable bathymodioline mussels to reconcile symbiotic tolerance with pathogen vigilance in deep-sea environments.

The holobiont perspective, retained here in the physiological sense introduced in Section 1, provides a useful framework for considering the immune biology of *Bathymodiolus azoricus*. Within the gill epithelium, intracellular chemosynthetic symbionts coexist with host cells that remain continuously exposed to environmental microorganisms, creating a physiological requirement to accommodate beneficial bacteria while retaining the capacity to respond to potentially harmful microbes [38,39,40]. In *B. azoricus*, previously reported ex vivo gill experiments and spatial-expression analyses provide evidence that microbial sensing and immune-gene expression are regulated at both local and tissue levels [13]. These observations form the basis for the host–symbiont–pathogen triad developed here as an integrative framework rather than as a single demonstrated molecular pathway (Figure 2). The working hypothesis introduced in Section 1 is developed empirically in the remainder of this section.

The gill occupies a strategic position at the interface between the external environment and internal body compartments, where exposure to microorganisms is unavoidable during respiration and feeding. In *B. azoricus*, gill tissue is therefore not merely respiratory or nutritional but also immunologically responsive. Experimental studies have documented transcriptional modulation of genes involved in microbial recognition, antioxidant defense, and antibacterial responses in gill tissue, while circulating hemocytes provide complementary phagocytic and immune functions [13,23]. The gill thus represents a site at which local tissue-associated sensing and systemic immune components can interact. Comparable transcriptomic responsiveness of the gill to *Vibrio* infection has been demonstrated in *Mytilus galloprovincialis* [42], providing independent bivalve context for the immunological role of this organ.

Previously reported ex vivo experiments used freshly dissected *B. azoricus* gill fragments exposed to enriched preparations of methanotrophic and thiotrophic endosymbionts and to *Vibrio parahaemolyticus*, under conditions with or without hemocyte-free hemolymph serum [13]. Exposure to the endosymbiont preparations in the absence of hemolymph produced a general increase in the expression of immune-recognition genes, including C-type lectin- and LPS-binding-protein-related transcripts, demonstrating that gill tissue itself is capable of responding transcriptionally to microbial stimuli [13]. When hemolymph serum was added to gill preparations exposed to endosymbionts, expression of these recognition genes decreased. In contrast, the presence of hemolymph increased immune-gene expression in gill preparations exposed to *V. parahaemolyticus* or sterile-seawater controls [13]. These experiments therefore provide evidence that soluble hemolymph constituents modify local gill immune responses in a stimulus-dependent manner, with symbiont-associated and non-symbiotic bacterial exposures producing different transcriptional outcomes.

This distinction is important because it suggests that microbial discrimination in *B. azoricus* cannot be reduced to receptor recognition by the gill epithelium alone. Rather, the ex vivo results are consistent with a layered system in which tissue-associated microbial sensing is further shaped by hemolymph-derived humoral factors [13]. The identity of the serum components responsible for this effect remains unresolved, as does the molecular mechanism by which they modify PRR-associated transcription. PGRPs provide one relevant candidate recognition system because multiple PGRP genes are expressed in *B. azoricus* gill tissue and have been specifically discussed in relation to symbiosis [43], but direct causal links between individual PRRs and the serum-dependent effects observed ex vivo have not yet been demonstrated.

Spatial analysis provided a second level of organization. Different regions of the *B. azoricus* gill were examined along the anterior–posterior axis, including the posterior budding zone, the youngest region of the growing gill. Immune-associated gene expression was markedly lower in this posterior region than in more mature gill sections [13]. The original interpretation suggested that this reduced transcriptional activity might provide an immune-permissive environment for symbiont acquisition [13]. However, subsequent ultrastructural work across bathymodioline mussels demonstrated that gill growth zones are initially aposymbiotic and that symbionts colonize newly formed filaments only after their formation [38].

**Figure 3:**
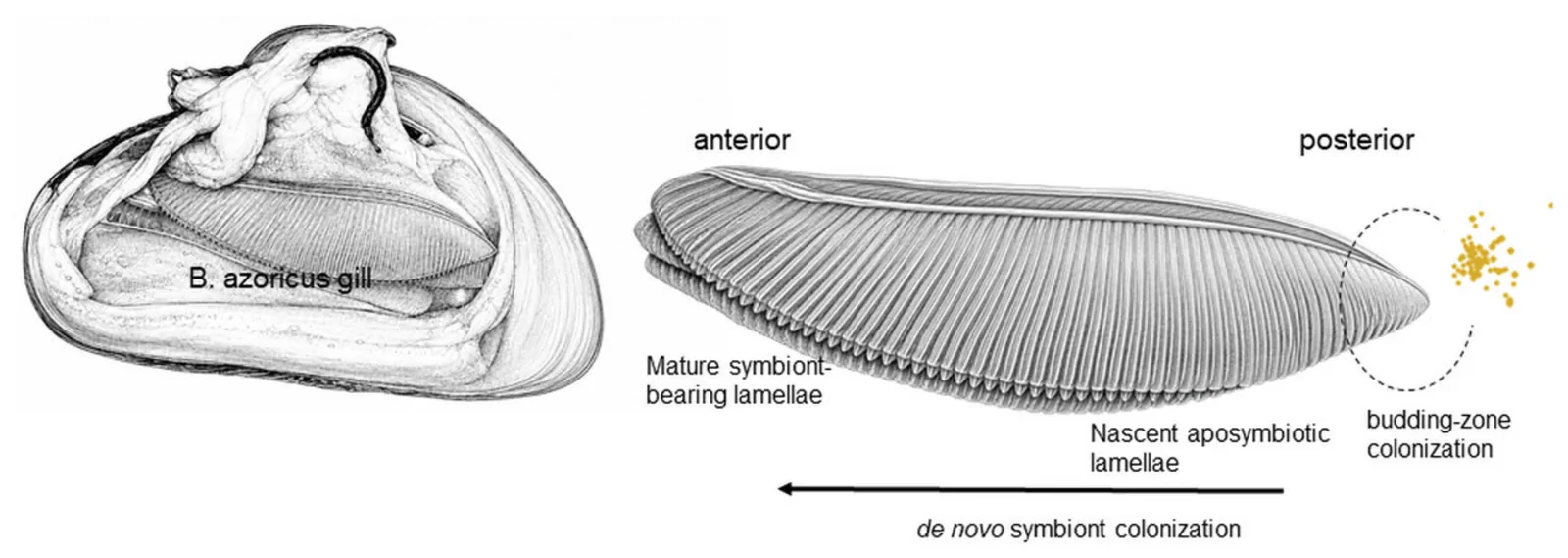
Anterior–posterior symbiont colonization gradient along the *B. azoricus* gill. Newly formed lamellae in the posterior budding zone are initially aposymbiotic, whereas symbiont abundance increases as filaments mature away from the growth zone. This conceptual gradient is consistent with ultrastructural observations showing that symbionts are absent from newly forming gill tissue and colonize filaments only after their formation [38]. The figure illustrates a developmental sequence and does not imply that reduced immune activity in the budding zone has been demonstrated to cause symbiont acquisition.

In light of those developmental observations, we consider tissue maturation an important alternative explanation for the *B. azoricus* spatial-expression pattern. Lower immune-gene expression in the budding zone may reflect the immature state of newly generated tissue rather than active immune suppression specifically imposed to allow symbiont entry. As filaments mature and become colonized, symbiont abundance and expression of immune-associated genes may increase together. The existing data do not establish causality or temporal order, and therefore do not distinguish definitively between developmental maturation, active symbiont-mediated immune modulation, or contributions from both processes [13,38] (Figure 3). This reassessment has implications beyond the present dataset: related interpretive claims elsewhere in the bathymodioline literature that rest on an actively permissive posterior microenvironment, rather than on its developmental immaturity, may likewise warrant reconsideration.

The mechanisms underlying the observed spatial transcriptional pattern remain unresolved. Developmental stage is an obvious candidate because the posterior budding zone consists of newly generated tissue [13,38], but additional factors—including differences in epithelial cell composition, hemocyte access, microbial exposure, or local signaling—may also contribute. At present, there is insufficient direct evidence to assign the zonation specifically to differential TLR, NF-κB, or cytokine-like pathway activity. These pathways remain plausible targets for future spatially resolved analyses rather than established drivers of the anterior–posterior gradient.

Temporal organization provides an additional angle that should be considered when interpreting gill transcription in *B. azoricus*. In situ temporal transcriptomics at Lucky Strike showed that 7.4% of the transcriptome oscillated within a 10.4–14.4 h range and 1.1% showed a 12.4 h period, with tidal cycles predominating in situ, whereas daily rhythms became more prominent after laboratory acclimation [44]. These findings do not invalidate controlled ex vivo challenge results, but they show that sampling time and environmental periodicity can contribute substantially to baseline transcriptional state and should therefore be considered when interpreting spatial or challenge-associated expression patterns.

A separate line of *B. azoricus* evidence shows that host immune responses and symbiont transcriptional activity can change concurrently during whole-animal bacterial challenge. In experiments using *Vibrio diabolicus*, mussels from the Menez Gwen and Lucky Strike vent fields exhibited time-, tissue-, and site-dependent changes in immune-associated genes involved in recognition, signal transduction, stress responses, and effector functions [45]. The genes examined included Carcinolectin, Serpin-2, SRCR, immune-responsive genes, receptor tyrosine kinase, TLR2, NF-κB, HSP70, and ferritin [45]. The two vent populations also differed in the temporal profiles of these responses, suggesting that environmental history and physiological state may contribute to differences in immune responsiveness.

Symbiont-associated metabolic genes, including ALDH, carbonic anhydrase, Calvin–Benson–Bassham-cycle components, methanol dehydrogenase, methane monooxygenase, and SOXB, were likewise transcriptionally modulated during acclimatization and bacterial challenge [46]. These observations show that host immune transcription and symbiont metabolic activity change within the same physiological context, although they do not by themselves demonstrate a direct causal regulatory interaction between the two [46]. The results nevertheless reinforce the view that the immune state of *B. azoricus* cannot be interpreted independently of the condition and abundance of its chemosynthetic partners.

Taken together, the available *B. azoricus* evidence supports a view of the gill as both a nutritional symbiotic organ and an immunologically responsive interface [13,45,46]. Ex vivo experiments demonstrate local microbial responsiveness that is further modified by hemolymph-associated factors [13], while spatial-expression analyses reveal marked anterior–posterior differences in immune-gene expression [13]. Independent developmental observations show that newly formed gill tissue is initially aposymbiotic and becomes colonized after filament formation [38]. These findings justify considering immune regulation in the gill as spatially structured, but they do not yet establish the molecular mechanism responsible for that structure. The host–symbiont–pathogen triad proposed here integrates four components observed at this interface: local microbial sensing, systemic humoral modulation, symbiont colonization, and gill development. What remains unresolved is the cause of the observed zonation: whether it reflects developmental maturation, active symbiont-mediated immune modulation, or a combination of both. This pattern is consistent with the working hypothesis proposed in Section 1, though direct experimental discrimination between developmental and symbiont-mediated contributions to gill zonation remains outstanding.

## 6. Symbiont Modulation of Host Immunity Across the Bathymodiolinae

The *B. azoricus* gill-zonation framework can be considered alongside a growing body of comparative transcriptomic, genomic, ultrastructural, and cell-biological work from other bathymodioline mussels. Studies in *Bathymodiolus japonicus* and *Gigantidas/Bathymodiolus platifrons*, in particular, have identified candidate mechanisms of symbiont acquisition and provided experimental or transcriptomic evidence for aspects of post-engulfment retention, digestion, and bacteriocyte turnover [17,19,20,47,48]. Because these mechanisms have not been demonstrated uniformly across the subfamily, the following sections retain explicit species attribution and distinguish functional evidence from genomic or transcriptomic inference.

### 6.1. Symbiont Acquisition: From Environment to Bacteriocyte

Symbiont acquisition begins at the gill surface, where epithelial cells encounter environmentally acquired bacteria [38,41]. Comparative genomic and transcriptomic evidence from Zheng et al. [41] points to coordinated changes on both sides of this interaction. On the host side, a mucin-5AC-like gene shows evidence of positive selection, consistent with modification of the host mucus layer during symbiont acquisition, and caveolin-related endocytic machinery has been implicated in microbial uptake at the epithelial surface. On the symbiont side, prtC, a mucin-digesting enzyme, shows both positive selection and elevated expression, consistent with a role in penetrating the host mucus barrier. These observations are consistent with modification of the epithelial interface from both host and symbiont sides during acquisition, but they do not demonstrate that either host mucin dynamics or symbiont-derived mucin digestion alone constitutes a symbiont-specific entry mechanism, and the two processes should not be conflated as a single mechanism. Direct cell-biological work in *B. japonicus* further showed that gill epithelial cells can engulf both symbiotic and non-symbiotic bacteria, indicating that microbial selectivity need not occur exclusively at the initial uptake step [17]. In that species, important discrimination emerges after engulfment through differential phagosome processing, as discussed in Section 6.2. More broadly, mucin-rich epithelial interfaces can contribute simultaneously to microbial tolerance and resistance, although this principle has been established mainly in other animal systems and does not demonstrate the *Bathymodiolus* acquisition mechanism [49].

Comparative genomic analysis of *B. platifrons* identified TLR13 as an expanded and strongly gill-expressed pattern-recognition receptor, together with expanded syndecan and protocadherin gene families that were proposed as candidate components of symbiont recognition and uptake [48]. Subsequent functional work in *G. platifrons* strengthened the evidence for a microbial-recognition role of TLR13: recombinant GpTLR13 was shown to bind LPS and particularly strongly to its lipid A component, while also showing binding to LTA [50].

However, these experiments did not demonstrate that GpTLR13 mediates the cellular uptake of methane-oxidizing symbionts. Thus, TLR13 has functional support as a microbial-recognition receptor in *G. platifrons*, whereas its proposed role as an apical gatekeeper for symbiont internalization remains hypothetical [48,50]. Syndecan and protocadherin likewise remain candidate uptake-associated factors based primarily on genomic expansion and expression evidence [48].

Importantly, neither the TLR13-mediated recognition mechanism nor the proposed TLR13–syndecan–protocadherin uptake model has been demonstrated in *B. azoricus*. Piquet et al. [51] discussed this model as potentially relevant to *B. azoricus*, but this represents extrapolation from comparative bathymodioline evidence rather than independent functional validation. By contrast, multiple PGRPs have been identified and expressed directly in *B. azoricus* gill tissue [43], providing species-specific evidence for candidate microbial-recognition components potentially involved in host–symbiont interactions.

Comparative evidence from *B. septemdierum* further indicates that PGRP expression can be spatially partitioned among gill cell types. Ikuta et al. identified a short-type PGRP expressed predominantly in asymbiotic goblet mucous cells and a long-type PGRP expressed in symbiont-bearing bacteriocytes, suggesting that distinct PGRP family members may participate in different aspects of microbial recognition at the gill interface [52].

**Figure 4:**
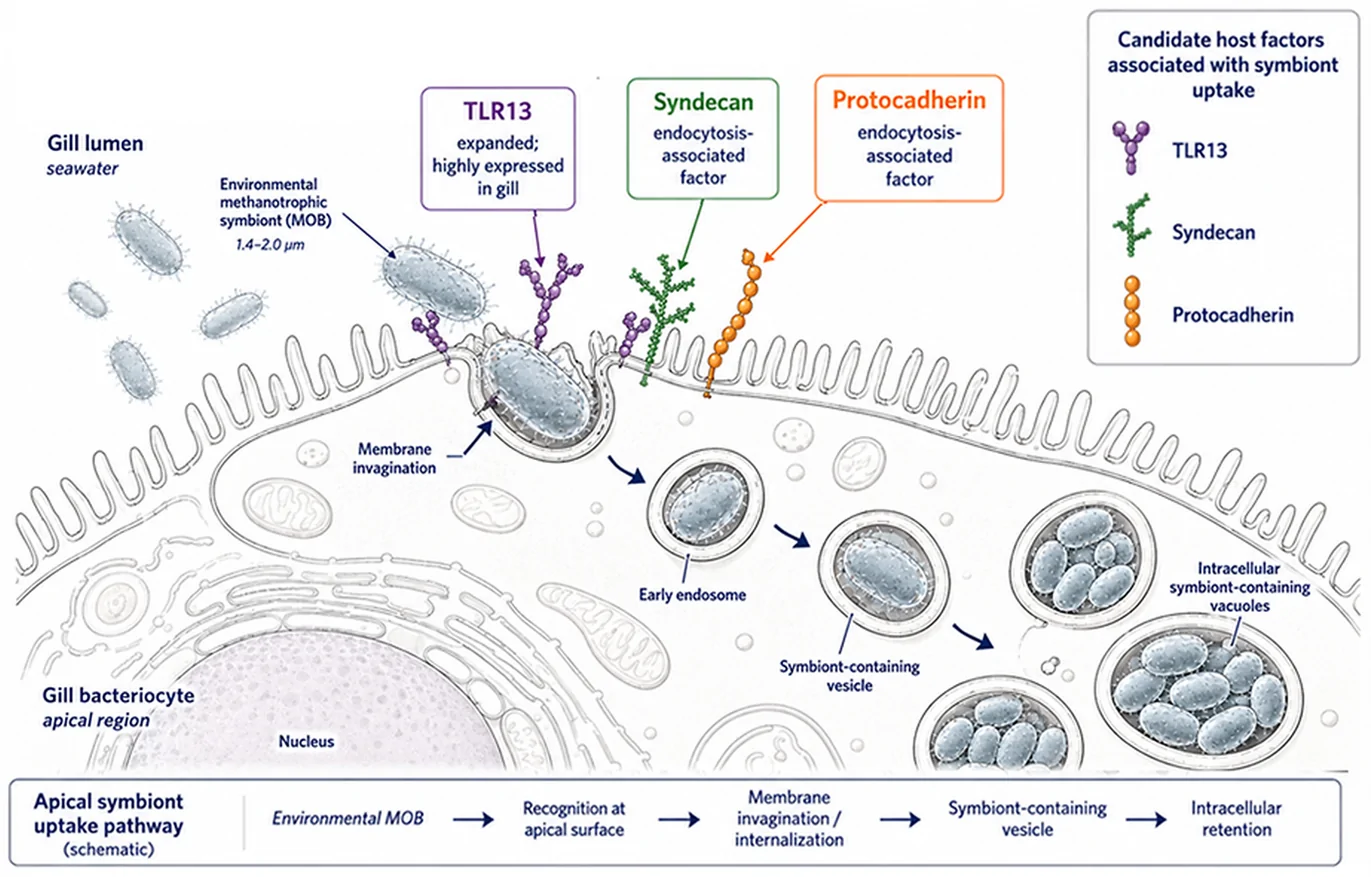
Schematic representation of a candidate endocytic mechanism for apical symbiont recognition and uptake in bathymodioline gills. Comparative genomic analysis in *B. platifrons* identified expansion and strong gill expression of TLR13, syndecan, and protocadherin gene families, which were proposed as candidate components of symbiont recognition and uptake [48]. Subsequent functional work in *G. platifrons* demonstrated binding of recombinant GpTLR13 to LPS, with particularly strong binding to its lipid A component, as well as binding to LTA [50], providing functional evidence for microbial ligand recognition but not for symbiont internalization. The participation of TLR13, syndecan, and protocadherin in a coordinated uptake mechanism therefore remains hypothetical, and this mechanism has not been demonstrated in *B. azoricus*. In *B. azoricus*, multiple PGRPs are expressed in gill tissue [43], providing species-specific evidence for *candidate microbial-recognition components potentially involved in host–symbiont interactions*. Arrows indicate the proposed direction of progression from apical microbial recognition through membrane invagination/internalization to formation of a symbiont-containing vesicle and subsequent intracellular retention; they represent a conceptual sequence rather than experimentally demonstrated causal transitions. The molecular sequence illustrated here should consequently be regarded as conceptual rather than as a directly demonstrated uptake pathway.

A complementary level of cellular resolution was obtained in *G. platifrons* by Wang et al., who enriched symbiont-bearing bacteriocytes and compared their transcriptomic profiles with those of whole gill tissue and the associated methane-oxidizing symbiont. The analysis revealed distinct bacteriocyte-associated expression patterns involving microbial-recognition, endocytic, lysosomal, and metabolic functions, while also indicating that non-bacteriocyte regions of the gill contribute to maintenance of the host–symbiont association [53]. These findings reinforce the view that symbiosis-related regulation is distributed across different cellular compartments of the gill rather than being restricted to bacteriocytes alone (Figure 4).

### 6.2. Cellular Selectivity and Differential Phagosome Processing

Direct cell-biological studies in *B. japonicus* have provided the clearest experimental evidence to date for post-engulfment microbial discrimination in a bathymodioline mussel. Gill epithelial cells were shown to internalize both symbiotic and non-symbiotic bacteria, including dead symbionts [17]. Exogenous bacteria subsequently entered acidifying, esterase-active digestive compartments, whereas resident symbionts persisted in vacuolar compartments showing minimal acidification and reduced esterase activity [17]. These observations indicate that microbial identity is not determined solely at the point of uptake but can be resolved through differential intracellular processing. For mechanistic context, canonical phagosome maturation involves progressive acidification and fusion with lysosomal compartments [54].

Subsequent work identified mTORC1 as a regulator of this process in *B. japonicus*. Nutritional signals associated with intracellular symbionts influence phagosome digestion, linking symbiont metabolic status to host retention or degradation [19,20]. This represents direct mechanistic evidence in *B. japonicus* and provides an important comparative model for the broader tolerance-versus-clearance problem considered in *B. azoricus*. However, the upstream signals controlling symbiosome maturation and the extent to which the same mechanism operates in other bathymodiolines remain unresolved.

### 6.3. Nutritional Strategies: Farming and Milking

Symbiont retention ultimately serves the nutritional economy of the host. Two non-exclusive models have been used to describe nutrient acquisition from intracellular symbionts in bathymodioline mussels. Farming refers to the digestion of symbiont cells and subsequent assimilation of their biomass and has direct ultrastructural and lysosomal support in several bathymodioline studies [38,51,55]. Milking, by contrast, refers to the non-destructive transfer of metabolites from living symbionts to host cells and remains more difficult to demonstrate directly; it should therefore be regarded as a proposed or inferred mode of nutrient transfer rather than an equivalently established mechanism [41,51].

The relative importance of these strategies is likely to vary with host nutritional state, symbiont physiology, and environmental substrate availability. Under starvation, increased symbiont digestion and gill remodeling are consistent with greater reliance on destructive nutrient extraction, whereas stable symbiosis may favor metabolite transfer from living symbionts [51]. These interpretations remain physiologically plausible but should not be taken to imply that the relative contributions of farming and milking have been quantitatively resolved in *B. azoricus*.

### 6.4. mTORC1, Lysosomal Dynamics, and Candidate Regulatory Pathways

Lysosomal regulation is central to the retention and digestion of intracellular symbionts, but the degree of mechanistic resolution differs substantially among candidate regulators. In *B. japonicus*, mTORC1 has been functionally implicated in controlling phagosome digestion in response to symbiont nutritional status [19]. This provides direct evidence linking nutrient sensing to intracellular microbial processing. More broadly, lysosomes are dynamic metabolic-signaling organelles that integrate nutrient sensing, mTORC1 activity, autophagy and lysosomal homeostasis [56,57].

Complementary evidence from *B. platifrons* links symbiont abundance with lysosome-associated host machinery. Yu et al. found that vesicle-associated membrane protein (VAMP) expression was associated with methane-oxidizing-symbiont-bearing regions of the gill and that both VAMP expression and methanotroph abundance declined after prolonged aquarium maintenance without methane supply [58]. These observations support an association between environmental substrate availability, symbiont persistence, and lysosome-related host processes, although they do not establish that VAMP itself determines symbiont retention or elimination.

TFEB is a conserved transcriptional regulator of lysosomal biogenesis and autophagy in metazoan systems [59] and is therefore a plausible candidate for regulating vesicular and lysosomal responses in bathymodioline bacteriocytes. However, its specific functional role in symbiont retention or digestion has not, to our knowledge, been experimentally demonstrated in *Bathymodiolus*. TFEB should consequently be treated as a candidate regulatory factor rather than as an established component of the mTORC1-controlled symbiosis mechanism.

Cathepsins and other lysosomal hydrolases provide a more directly supported downstream component of the digestive machinery. Their presence is supported in multiple bathymodioline transcriptomic datasets, including the *B. azoricus* gill transcriptomes discussed in Section 4 [11], while direct cellular studies in *B. japonicus* demonstrate differential acidification and digestive activity between resident-symbiont compartments and phagosomes containing exogenous bacteria [17,19]. Together, these observations support a lysosome-centered model of symbiont processing while leaving many upstream regulatory connections unresolved.

The organization of symbiont-containing vacuoles is itself variable in *B. azoricus*. Ultrastructural analyses have shown that sulfur- and methane-oxidizing symbionts may occupy separate or shared vacuolar compartments depending on the vent population examined, with individuals from Menez Gwen showing stronger segregation than those from Lucky Strike [51]. This indicates that symbiosome architecture is not necessarily fixed across populations and may vary in association with local physiological or environmental conditions. Whether this variation reflects host genotype, local geochemical or nutritional conditions at each vent site, or differences in sample fixation and processing between studies cannot be determined from the available data and remains an open methodological as well as biological question.

### 6.5. Bacteriocyte Turnover, Gill Remodeling, and Starvation Plasticity

Bacteriocyte abundance and epithelial organization are dynamic rather than fixed. Developmental and cell-renewal studies in *Gigantidas/Bathymodiolus platifrons* indicate that bacteriocyte turnover, apoptosis, and proliferative cell populations contribute to maintenance of the symbiotic gill epithelium [38,47]. In *B. azoricus*, Piquet et al. [51] documented extensive ultrastructural remodeling during prolonged symbiont loss under aquarium starvation conditions. Symbiont-containing compartments progressively underwent lysosomal processing, bacteriocyte abundance declined, and the gill epithelium shifted toward a morphology more characteristic of a non-symbiotic filter-feeding state.

Comparative genomic analysis of *B. platifrons* also identified expansion of apoptosis-related gene families, including inhibitor-of-apoptosis proteins, with several members strongly expressed in the gill [48]. This genomic pattern suggests evolutionary remodeling of cell-death regulation in bathymodioline symbiosis, but does not by itself establish how apoptosis is controlled during symbiont maintenance or loss. Comparative analysis of the bivalve caspase family likewise reveals substantial lineage-specific complexity in programmed-cell-death machinery [60].

Earlier experimental work in *B. azoricus* similarly showed progressive loss of detectable symbionts during starvation and subsequent re-establishment of symbiont populations after renewed exposure to sulfide in the presence of symbiont-bearing mussels [61]. These findings demonstrate that symbiont abundance in *B. azoricus* is highly plastic and can recover after experimentally induced loss, while the gill undergoes substantial structural plasticity as symbiont abundance changes. Lifelong competence for symbiont acquisition, demonstrated more broadly across bathymodioline mussels [38], further emphasizes the dynamic nature of these horizontally transmitted associations.

**Figure 5:**
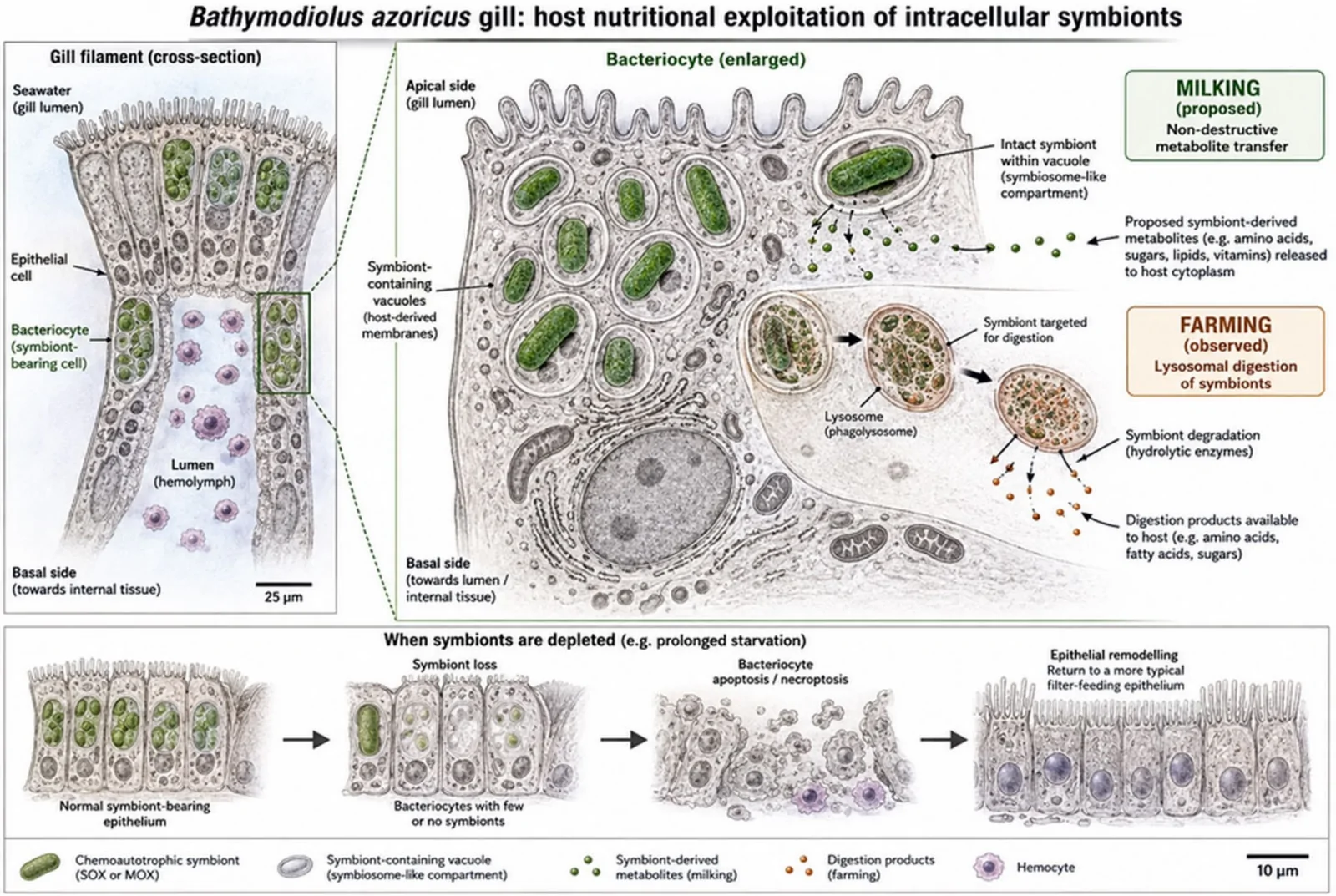
Nutrient acquisition and bacteriocyte remodeling in the *B. azoricus* gill. Host cells can obtain nutrients from intracellular symbionts through lysosomal digestion (“farming”), which has direct ultrastructural support, whereas continuous non-destructive metabolite transfer (“milking”) remains a proposed mechanism [51]. During prolonged starvation and symbiont loss, bacteriocyte abundance decreases and the gill epithelium remodels toward a less symbiotic, filter-feeding morphology [51]. In the lower sequence, large horizontal arrows indicate the progression observed during prolonged symbiont depletion/starvation, from a normal symbiont-bearing epithelium through symbiont loss and bacteriocyte apoptosis/remodeling toward a more typical filter-feeding epithelial organization. In the enlarged bacteriocyte, smaller arrows and dotted trajectories indicate the direction of the proposed non-destructive transfer of symbiont-derived metabolites (‘milking’) and the lysosomal digestion pathway through which symbiont biomass is degraded and nutrients become available to the host (‘farming’). The figure distinguishes directly observed cellular events from proposed nutrient-transfer mechanisms.

Piquet et al. [51] further proposed that a nutritionally deprived symbiont might become metabolically costly to the host and therefore become a target for digestion or elimination. This is an explicit hypothesis rather than a demonstrated switch from mutualism to parasitism and should be interpreted accordingly. Nevertheless, it provides a useful conceptual framework for considering how host cells might alter symbiont handling when the nutritional balance of the association changes (Figure 5).

### 6.6. Genomic and Transcriptomic Signatures of Immune-System Remodeling

Comparative genomic and transcriptomic studies have identified lineage-specific changes in immune-associated gene families across bathymodioline mussels, including variation in Toll-like receptors, C1q-domain-containing proteins, lectins, cathepsins, and genes involved in vesicular trafficking and lysosomal function [41,48,50,62,63]. Some studies report reduced diversity in selected PRR families together with expansion or positive selection in others [41,48,50,62,63]. These patterns are consistent with evolutionary remodeling of microbial-recognition and intracellular-processing systems in chemosymbiotic lineages, but they do not demonstrate that reduced PRR diversity itself causes immune tolerance.

Developmental transcriptomic work in *B. platifrons* further indicates stage-dependent differences in pathways associated with autophagy, lysosomal processing, and apoptosis [64]. These observations suggest that symbiont management may change during ontogeny, although transcriptomic enrichment should not be equated directly with demonstrated pathway activity. Comparative tissue analyses also show elevated expression of microbial-recognition, apoptosis-associated, and detoxification genes in symbiont-bearing gill relative to non-symbiotic tissues [65], reinforcing the specialized immunological and metabolic role of the gill.

The *B. azoricus*-specific comparison between the 2010 and 2024 gill transcriptomes is discussed separately in Section 4, where independent recovery of several immune-associated gene families provides additional transcriptomic support without implying functional equivalence to mechanisms demonstrated experimentally in other bathymodioline species.

### 6.7. MicroRNA-Mediated Immune Tuning

Post-transcriptional regulation by microRNAs adds another potential layer of immune modulation. In *G. platifrons*, gill profiling identified 459 microRNAs together with immune-associated transcriptional responses following challenge with enriched methane-oxidizing symbionts and non-symbiotic *Vibrio* [66]. Multiple PRRs, signaling components, and immune effectors showed stimulus- and time-dependent expression, accompanied by distinct microRNA profiles at 12 and 24 h after exposure [66].

These data are consistent with microRNA-mediated regulation contributing to differential host responses to symbiotic and non-symbiotic bacteria, although the individual regulatory interactions remain to be functionally validated. Comparable microRNA-level analyses have not yet been reported for *B. azoricus* and represent a clear target for future work.

### 6.8. Broader Evolutionary Context: Convergence and Diversity Across Chemosymbiotic Lineages

The mechanisms discussed above occur within a bathymodioline lineage whose evolutionary history substantially predates the modern hydrothermal-vent habitats in which many species are now found. Time-calibrated phylogenetic analyses place the origin of Bathymodiolinae and their association with sulfur-oxidizing symbionts in the Late Cretaceous, with subsequent radiation into vents and seeps during the Eocene–Oligocene interval [67]. Within the subfamily, symbiont complement remains diverse: *B. azoricus* and *B. puteoserpentis* maintain dual sulfur- and methane-oxidizing symbioses [8], whereas other species are dominated by a single major chemosynthetic symbiont type. This diversity cautions against assuming that a single immune-symbiosis mechanism applies uniformly across all bathymodiolines.

A potentially convergent pattern is seen outside Mollusca. The siboglinid tubeworm *Lamellibrachia luymesi*, which evolved chemosymbiosis independently of bathymodioline mussels, possesses an expanded Toll-like receptor repertoire and a putative TLR4-like pathway proposed to contribute to host–symbiont and pathogen interactions [68]. The recurrence of PRR-family expansion in independently evolved chemosymbiotic hosts is consistent with convergent selective pressures on microbial-recognition systems [48,68], although functional equivalence between the lineages has not been demonstrated. This parallel is offered as suggestive of convergent selective pressure rather than as direct evidence bearing on the mechanisms operating in *B. azoricus*.

Vesicomyid clams provide a contrasting example of symbiont transmission. Unlike the predominantly horizontal acquisition characteristic of bathymodioline mussels, vesicomyids are generally described as transmitting sulfur-oxidizing symbionts vertically through the female germ line. In some species, symbionts localize to the vegetal pole of the egg, providing direct evidence for maternal transmission [69]. Phylogenomic analyses nevertheless reveal occasional historical host-switching events among vesicomyid symbionts [70], showing that even systems dominated by vertical transmission can retain some evolutionary flexibility in partner acquisition.

This comparison highlights an unresolved question in *B. azoricus* which consists of determining the immediate source of symbionts that colonize newly formed gill tissue. Lifelong competence for symbiont acquisition is well supported across bathymodioline mussels [38], but the relative contribution of free-living environmental populations, locally released symbionts, or transfer among neighboring hosts remains incompletely resolved for *B. azoricus*. Transmission mode should therefore be treated as a dynamic ecological process rather than as a simple binary distinction between horizontal and vertical inheritance. This conceptual reframing, while useful, does not by itself resolve the practical challenge of identifying symbiont sources for individual experimental animals, which remains a methodological constraint on *B. azoricus* studies.

Taken together, the comparative literature does not support a single uniform bathymodioline immune–symbiosis mechanism. Instead, different species currently provide evidence at different biological levels: *B. azoricus* for hemolymph-dependent immune modulation and gill-level transcriptional zonation; *B. platifrons* for candidate uptake-associated genomic features; *B. japonicus* for experimentally resolved post-engulfment processing and mTORC1-dependent symbiont digestion; and *G. platifrons* (the same species as *B. platifrons*, referred to here by the name used in each respective source) for microRNA-associated and single-cell regulatory complexity. None of these mechanisms has yet been demonstrated comprehensively across multiple species. The resulting mosaic should therefore be treated as a comparative framework from which conserved principles can be hypothesized, rather than as proof that all bathymodioline mussels use the same molecular solution.

### 6.9. Alternative Explanatory Frameworks for Symbiont Accommodation

The synthesis developed above has emphasized an immunocentric perspective, in which spatially structured immune-gene expression, hemolymph-dependent modulation, and nutrient-sensitive phagosome processing are treated as components of a host–symbiont–pathogen triad. This framing is not the only plausible account of how bathymodioline mussels tolerate persistent intracellular symbionts, and three alternative or complementary explanatory principles deserve explicit consideration.

One alternative treats metabolic constraint, rather than immune regulation per se, as the primary driver of symbiont fate. Under this view, the mTORC1-dependent checkpoint demonstrated in *B. japonicus* [19,20] reflects a nutrient-sensing mechanism whose immunological correlates are downstream or incidental rather than causal, with symbiont retention or digestion determined principally by the host’s own nutritional economy and pattern-recognition signaling contributing comparatively little to outcome. Distinguishing this possibility from the immunocentric reading would require experiments that manipulate nutrient status while holding immune stimulus constant, which have not, to our knowledge, been performed in any bathymodioline species.

A second alternative holds that physical compartmentalization is sufficient by itself to prevent immune engagement with intracellular symbionts, without requiring active suppression or modulation of immune-gene expression. Sequestration of symbionts within specialized vacuolar compartments [51] could in principle isolate bacterial ligands from cytoplasmic or membrane-associated pattern-recognition receptors regardless of the transcriptional state of the surrounding cell. This possibility is difficult to evaluate with the bulk-tissue transcriptomic data available for *B. azoricus* (Section 9.1); it predicts that tissue-level immune-gene expression differences reflect cellular composition rather than a regulated response, a prediction that single-cell or single-nucleus approaches could test directly.

A third alternative is that symbionts themselves actively dampen host immune responses, analogous to mechanisms described in other host–microbe associations in which microbiota-derived signals shape innate immune tone [40]. No direct evidence for symbiont-derived immunosuppression currently exists in *B. azoricus* or other bathymodiolines, and this possibility is offered here as a hypothesis rather than as an established mechanism. This hypothesis should be considered against a broader comparative background in which innate immune components can participate in establishing and maintaining beneficial bacterial symbioses in other invertebrates [71,72].

These alternatives are not mutually exclusive, and elements of each may operate simultaneously at different biological scales. We have not attempted to adjudicate among them, because the cell-resolved and experimentally manipulated data necessary to do so are not yet available for *B. azoricus*. We flag them explicitly, however, so that the host–symbiont–pathogen triad developed in this review is read as one candidate organizing framework among several plausible accounts, rather than as the only framework consistent with current evidence.

## 7. Toward an Integrated Model: The Holobiont as a Spatially Structured Immune System

### 7.1. Three Pillars of the Model

The preceding sections identify three complementary levels at which host–microbe interactions can be considered in bathymodioline gills. The first is the *host–symbiont–pathogen triad*, which describes the spatial and physiological coexistence of intracellular nutritional symbionts, host epithelial cells, and potentially harmful environmental microorganisms at the gill interface (Section 5). The second comprises *immune and metabolic signaling networks*, including pattern-recognition pathways identified at the transcriptomic level in *B. azoricus* and nutrient-sensitive intracellular processing demonstrated experimentally in *B. japonicus* (Section 3, Section 4 and Section 6). The third is the *cellular and tissue architecture of symbiosis*, encompassing bacteriocyte physiology, lysosomal processing, symbiont acquisition and loss, epithelial remodeling, and anterior–posterior differences in gill immune-gene expression (Section 5 and Section 6). These levels are integrated here as a conceptual framework, while retaining the distinction between mechanisms directly demonstrated in individual species and relationships that remain inferential.

### 7.2. Spatial Architecture: From the General Triad to Gill-Level Heterogeneity in B. azoricus

The general triad introduced in Section 5 describes the gill as a compartmentalized host–microbe interface: intracellular symbionts reside within bacteriocytes, host cytoplasm and vesicular compartments regulate their persistence, and environmental microbes encounter the apical epithelial surface [8,11]. In *B. azoricus*, this cellular organization is accompanied by an additional tissue-level feature: immune-associated gene expression differs along the anterior–posterior gill axis, with lower expression in the posterior budding region than in mature anterior tissue [13]. These transcriptional differences provide evidence that immune state is spatially heterogeneous within the organ, although their functional significance remains unresolved. This spatial heterogeneity is the principal empirical basis for the working hypothesis proposed in Section 1: that immune competence in the gill is organized primarily along developmental rather than purely symbiont-driven lines.

This tissue-level pattern can be considered alongside the cellular checkpoint demonstrated in *B. japonicus*, where mTORC1 regulates phagosome digestion according to symbiont nutritional status [19,20]. The two observations occur at different biological scales and in different species: one concerns spatial variation in gene expression across the *B. azoricus* gill, whereas the other concerns intracellular regulation of microbial digestion within *B. japonicus* bacteriocytes. Whether they represent independent adaptations, evolutionarily related regulatory principles, or different levels of a more broadly conserved mechanism is currently unknown. Establishing that relationship will require cellularly and spatially resolved functional studies in *B. azoricus*.

### 7.3. Signaling Networks and Cellular Processes

Pattern-recognition receptors provide the molecular entry point for innate microbial sensing. The *B. azoricus* gill transcriptomes contain homologs of TLRs, PGRPs, lectins, scavenger receptors, MyD88, TRAF6, IRAK-family proteins, NF-κB and other canonical innate-immune components [11]. Comparative studies likewise reveal lineage-specific expansion, contraction, and positive selection in several immune-associated gene families across the Bathymodiolinae [41,48,50,62,63,64,65]. These observations establish the presence and evolutionary diversification of candidate signaling machinery, but they do not demonstrate that a complete canonical signaling cascade operates identically in *Bathymodiolus*. Functional studies in *Crassostrea gigas* have shown interactions of oyster IKKα/β proteins with MyD88 and TRAF6, providing comparative bivalve support for TLR-associated signaling involving these components [73].

**Figure 6:**
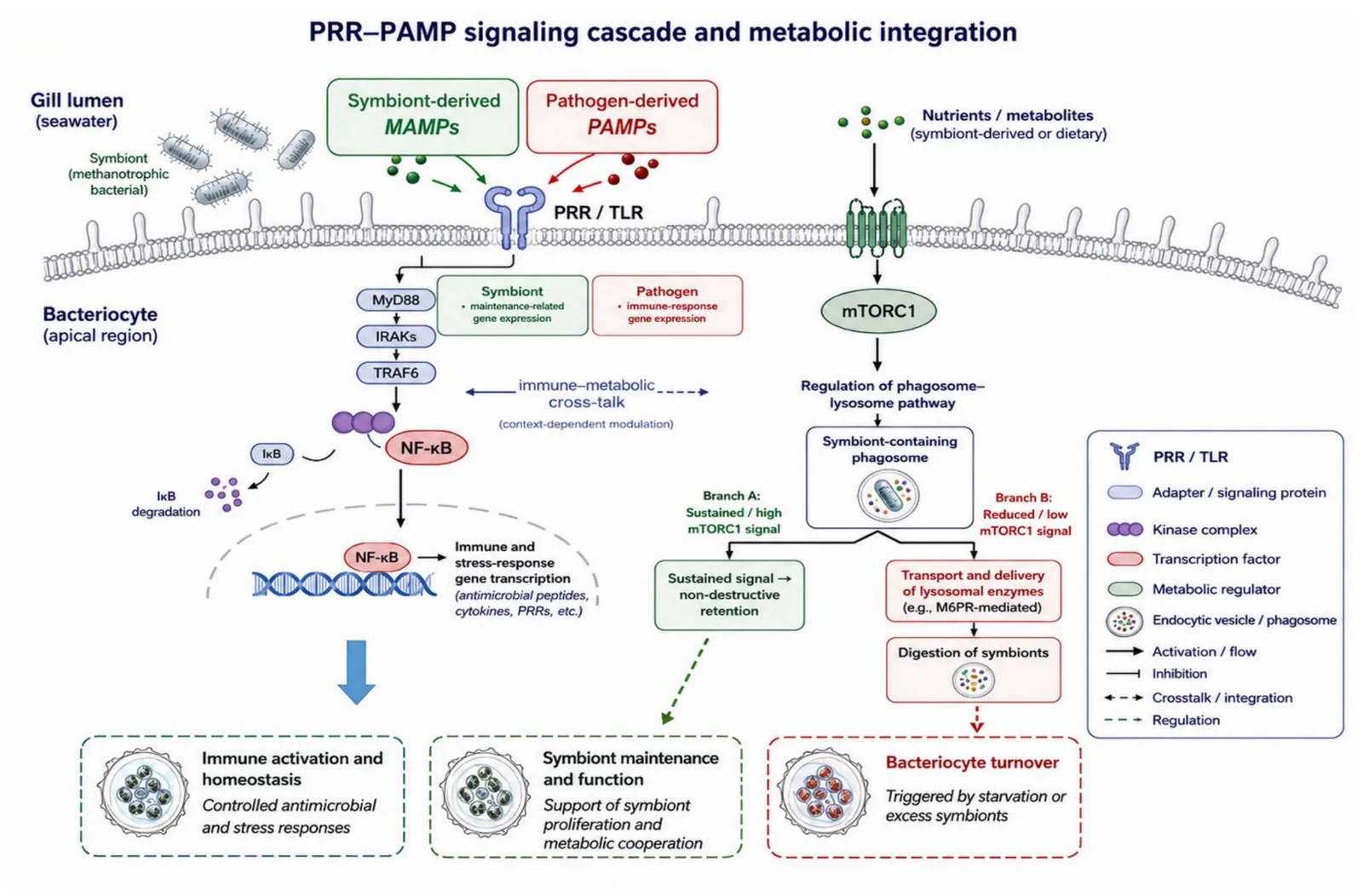
Conceptual integration of microbial recognition, hemolymph-dependent immune modulation, and nutrient-sensitive symbiont processing in bathymodioline gills. The left branch illustrates a canonical PRR-associated MyD88–IRAK–TRAF6–NF-κB signaling framework and is included for mechanistic context; this complete cascade has not been functionally demonstrated in Bathymodiolus [74]. In *B. azoricus*, ex vivo gill experiments provide species-specific evidence that soluble hemolymph factors modify immune-recognition gene expression in a microbial-stimulus-dependent manner [13]. The right branch summarizes mTORC1-dependent regulation of phagosome fate demonstrated specifically in *B. japonicus* [19,20]. Following formation of the symbiont-containing phagosome, the pathway is shown as two explicit, signal-dependent branches rather than a single linear outcome: under a sustained or high mTORC1 signal (Branch A), the phagosome is retained without destructive processing, consistent with non-destructive nutrient transfer (“milking”); under a reduced or low mTORC1 signal (Branch B), lysosomal enzymes are transported and delivered to the phagosome (e.g., via M6PR-mediated trafficking) and the symbiont is digested. This direction-dependence follows the mechanism reported by Tame et al. [19]. The dashed connection between immune and metabolic branches denotes conceptual integration rather than experimentally demonstrated molecular crosstalk. Symbiont maintenance and bacteriocyte turnover are shown as alternative, signal-dependent outcomes rather than sequential steps of a single pathway, and neither is a direct terminal consequence of NF-κB activation, which is linked only to the immune-activation outcome on the left [51].

For conceptual purposes, Figure 6 therefore uses the canonical MyD88–IRAK–TRAF6–NF-κB pathway as a reference framework for PRR-associated signaling. In established innate-immune systems, activation of this pathway can induce transcriptional programs associated with innate immune and inflammatory responses [74]. Apoptosis is not represented as an obligatory terminal outcome of NF-κB signaling; rather, cell death and bacteriocyte turnover are treated as distinct processes associated with physiological stress, symbiont loss, and tissue remodeling [51].

A separate metabolic branch is supported by direct work in *B. japonicus*, where mTORC1 regulates phagosome digestion in response to symbiont nutritional status [19,20]. This pathway links nutrient sensing to intracellular symbiont retention or degradation but has not been shown to lie downstream of PRR–NF-κB signaling. Similarly, TFEB (Section 6.4) is retained only as a candidate lysosomal regulator and should not be interpreted as a demonstrated component of the *Bathymodiolus* symbiosis pathway.

The integration of immune recognition and metabolic symbiont processing proposed here is therefore conceptual. It reflects the biological requirement for a bacteriocyte to coordinate microbial sensing, intracellular trafficking, nutrient acquisition, and cellular homeostasis, while acknowledging that direct molecular crosstalk between the PRR–NF-κB and mTORC1–lysosomal branches has not yet been demonstrated in bathymodioline mussels (Figure 6).

### 7.4. Holobiont Resilience: Plasticity Across Biological Scales

Considered together, the available evidence points to regulation operating across multiple biological scales. Comparative genomic and transcriptomic studies indicate lineage-specific remodeling of immune and lysosomal gene repertoires [41,48,50,62,63,64,65]; cellular work demonstrates differential intracellular processing of symbiotic and non-symbiotic bacteria in *B. japonicus* [17,19]; ultrastructural studies show extensive bacteriocyte and gill remodeling during symbiont loss in *B. azoricus* [51,61]; and spatial-expression analyses indicate anterior–posterior heterogeneity in *B. azoricus* gill immune-gene activity [13]. These processes collectively support the view that bathymodioline symbioses are physiologically plastic rather than static.

Consistent with the physiological definition of holobiont adopted in Section 1, the holobiont model proposed here does not assume that all of these mechanisms are controlled by a single regulatory circuit or are conserved identically across species. Instead, it provides a multiscale framework in which microbial recognition, intracellular symbiont processing, host nutrition, cell turnover, and tissue development can be considered together. The central unresolved question is how these levels are coordinated mechanistically within a single species—particularly *B. azoricus*—and whether the cellular checkpoints identified in other bathymodiolines operate alongside the tissue-level zonation described here. This question corresponds directly to the working hypothesis proposed in Section 1: if immune-gene expression is found to track maturation state independently of symbiont occupancy, spatial and developmental compartmentalization emerges as the dominant explanatory principle; if occupancy itself modulates expression at matched maturation stages, an additional layer of symbiont-mediated regulation would need to be invoked.

## 8. Prospective Biotechnological Relevance

The mechanisms reviewed here are primarily of fundamental biological interest, but they may also have longer-term relevance beyond deep-sea symbiosis research. Bathymodioline mussels provide an unusual natural model in which an animal host maintains dense populations of intracellular beneficial bacteria within an immunologically responsive epithelium while retaining the capacity to respond to potentially harmful microorganisms. Understanding how microbial recognition, intracellular trafficking, nutrient sensing, and tissue remodeling are coordinated in such a system may therefore inform broader questions in host–microbiome biology, particularly the mechanisms that provide persistent microbial accommodation without generalized loss of immune competence. More broadly, recognition of animals as integrated host-microbial systems has reshaped modern host biology [75].

From this perspective, the most immediate translational value of *Bathymodiolus* lies not in any currently identified therapeutic molecule, but in the biological principles revealed by the symbiosis. The differential handling of symbiotic and non-symbiotic bacteria, the contribution of soluble hemolymph factors to gill immune responses in *B. azoricus*, and the nutrient-sensitive regulation of intracellular symbiont digestion demonstrated in *B. japonicus* provide comparative models for studying how animal tissues distinguish, retain, or eliminate microbial partners [13,17,19,20]. Such systems may ultimately contribute to a more general understanding of mucosal immunity, intracellular microbial persistence, and host–microbiome homeostasis, although direct parallels with vertebrate or human microbiomes should not be assumed.

A second potential area of relevance concerns marine animal health. The immune and symbiosis-associated markers identified in *B. azoricus* and related bathymodiolines—including PRRs, lysosomal proteins, hemolymph-associated modulators, and symbiont metabolic indicators—could provide candidate biomarkers for assessing host physiological state, symbiosis stability, or responses to microbial challenge. In principle, comparable approaches may prove useful in aquaculture and conservation settings where host health is closely linked to associated microbiota. However, no such applications have yet been validated from *B. azoricus*, and their utility will depend on functional characterization of the relevant molecules and on determining which regulatory principles are conserved beyond deep-sea chemosymbiotic mussels.

The most defensible biotechnological significance of the host–symbiont–pathogen triad is therefore conceptual rather than immediately translational. Bathymodioline mussels offer a naturally evolved system in which immune surveillance, microbial accommodation, intracellular nutrient acquisition, and tissue plasticity coexist within the same organ. Defining the molecular basis of that coexistence may identify regulatory principles of broader biological relevance, but translation into engineered microbiomes, disease models, or applied marine-health tools remains a future possibility rather than an established outcome. Even at this conceptual stage, the mechanisms reviewed here may be informative beyond *Bathymodiolus*: principles governing selective microbial tolerance at a mucosal-like epithelial interface are directly relevant to mucosal immunology in other systems, and a molecularly defined account of stable host-symbiont coexistence could usefully inform microbiome-engineering approaches that seek to maintain beneficial microbial populations without compromising host defense [76].

## 9. Methodological Caveats and Future Directions

### 9.1. Caveats of the Existing B. azoricus Datasets

Interpretation of the available *B. azoricus* molecular datasets requires consideration of the methodological differences among studies. The 2010 gill transcriptome that underlies a substantial part of the immune-gene catalog discussed in Section 4 was generated from a normalized, poly(A)-selected cDNA library using 454 pyrosequencing [11]. Because the cDNA library was normalized, the relative representation of reads or contigs in this dataset cannot be interpreted directly as a quantitative measure of the original transcript abundances or used to compare the abundance of different immune-gene families.

This limitation is particularly important when the 2010 resource is compared with later Illumina-based datasets generated using different library-preparation procedures, sequencing depths, assembly algorithms, and annotation pipelines. Differences in the number of recovered transcripts or members assigned to particular immune-gene families may reflect methodological effects as well as biological variation. The independent recovery of homologous immune-associated sequences across datasets therefore provides useful transcriptomic corroboration, but should not by itself be interpreted as evidence for differential expression, gene-family expansion, or functional conservation.

A second limitation concerns the experimental context in which much of the *B. azoricus* physiological and immunological work has been performed. Long-term aquarium maintenance at atmospheric pressure made controlled experimentation possible [12,16], but decompression, altered hydrostatic pressure, substrate availability, temperature, feeding conditions, and progressive changes in symbiont abundance can all influence host physiology. Results obtained during aquarium acclimatization should therefore be interpreted as responses under experimentally altered conditions rather than as exact representations of the intact vent environment. This caveat is particularly relevant to studies examining immune transcription, symbiont loss, and metabolic remodeling over extended periods [15,46,51,61]. Temporal sampling is an additional consideration: in situ gill transcriptomes show pronounced tidal rhythmicity, whereas daily rhythms become more prominent after laboratory acclimation [44]. Thus, sampling phase may contribute to expression differences independently of experimental treatment and should be controlled or recorded where possible.

Finally, many of the available immune-expression studies rely on bulk tissue. A gill fragment contains multiple epithelial and non-epithelial cell types, and transcriptional changes measured at the tissue level cannot automatically be assigned to bacteriocytes or another specific cellular population. This distinction is especially important for the anterior–posterior expression pattern discussed in Section 5: lower expression of immune-associated genes in the posterior budding zone may reflect differences in cellular composition, developmental state, transcriptional activity within the same cell type, or some combination of these factors. Current data do not distinguish between these possibilities.

### 9.2. Advancing the Holobiont Framework

Resolving cellular heterogeneity is therefore one of the most important next steps for *B. azoricus*. Single-cell or single-nucleus transcriptomics, complemented by spatial transcriptomics, in situ hybridization, immunolocalization, and spatial proteomics, could identify the cell populations responsible for the immune and metabolic expression patterns currently observed only at bulk-tissue level. The feasibility and value of this approach are demonstrated by recent single-nucleus RNA-sequencing of the *G. platifrons* gill, which resolved 13 cell types and provided a cellular framework for investigating symbiosis-associated differentiation and gill organization [18]. Applying comparable approaches to *B. azoricus* would provide a direct means of testing whether the anterior–posterior zonation described in Section 5 results from differences in cell-type composition, developmental maturation, distinct transcriptional states within related epithelial populations, or combinations of these processes.

Functional validation represents a second major priority. Candidate pattern-recognition receptors identified in *B. azoricus*, including PGRPs [43], could be examined through ligand-binding assays, cellular localization, targeted inhibition, or gene-expression perturbation. Comparative work in *G. platifrons* illustrates the value of this progression from sequence discovery to functional analysis: recombinant GpTLR13 was shown to bind LPS through its lipid A component [50], although its proposed role in symbiont uptake remains unproven. Similar experiments in *B. azoricus* would help distinguish receptors involved simply in microbial recognition from those participating specifically in symbiont accommodation.

The mTORC1-dependent control of phagosome digestion demonstrated in *B. japonicus* [19] represents another particularly important comparative hypothesis to test. At present, there is no evidence that the same intracellular checkpoint operates in *B. azoricus*. Pharmacological manipulation of nutrient-sensing and lysosomal pathways, combined with quantitative imaging of symbiont-containing compartments and measurements of symbiont viability or digestion, could provide an experimentally accessible first step. TFEB (Section 6.4), by contrast, should remain a candidate regulator until its expression, localization, and functional involvement are demonstrated directly in bathymodioline bacteriocytes.

Gene knockdown, RNA interference, or genome-editing approaches may ultimately provide causal tests of candidate pathways, but, to our knowledge, these methods are not yet routine in *Bathymodiolus* and should be regarded as longer-term methodological objectives rather than immediately available experimental tools. Establishing reliable primary-cell, organ-explant, or ex vivo gill systems may therefore be a more realistic intermediate route toward mechanistic manipulation.

A third priority is to integrate host and symbiont measurements within the same experiments. Temporal combinations of metatranscriptomics or metagenomics, metabolomics, quantitative microscopy, and host transcriptomics could determine how changes in environmental substrates, symbiont abundance, symbiont metabolic state, and host immune activity co-vary through time. Such approaches would be particularly valuable for distinguishing correlation from causal interaction in the concurrent host-immune and symbiont-metabolic responses observed during acclimatization and microbial challenge [15,45,46].

Finally, broader comparative sampling across the *Bathymodiolinae* is required. Current mechanistic understanding is distributed unevenly among species: *B. azoricus* provides evidence for hemolymph-dependent modulation and gill-level transcriptional heterogeneity; *B. japonicus* provides direct evidence for post-engulfment discrimination and mTORC1-dependent symbiont digestion [17,19]; and *B./G. platifrons* provides genomic, receptor-binding, microRNA, developmental, and single-cell evidence [18,48,50,62,64,66]. Comparative phylogenomics and equivalent functional assays across species will be necessary to determine which features represent conserved bathymodioline principles and which are lineage-specific adaptations.

### 9.3. Open Questions Specific to B. azoricus

Several important gaps remain specific to *B. azoricus*. One concerns the cellular organization of systemic immunity. Hemocytes clearly contribute to phagocytosis, oxidative responses, and soluble hemolymph-mediated modulation of gill immune expression [13,23], but the functional significance of the morphologically described hemocyte populations remains incompletely resolved. Modern cell-sorting, cytometric, transcriptomic, and imaging approaches could determine whether these populations represent functionally distinct immune-cell states and how they interact with the symbiotic gill.

A second gap concerns antimicrobial effectors. Evidence for mytilin in *B. azoricus* derives from homology-based detection and expression analysis of a mytilin-related transcript rather than from cloning and complete characterization of a species-specific gene [23]. A big-defensin homolog is also represented at the sequence level (BDEF_BATAZ; HM756150) and was included in comparative analysis with oyster big defensins [34], but its expression and antimicrobial function have not been characterized in *B. azoricus*. The diversity, tissue distribution, inducibility, and biochemical activity of AMP families in this species therefore remain substantially unresolved.

A third major question is whether the intracellular nutrient-sensitive checkpoint demonstrated in *B. japonicus* also operates in *B. azoricus*. In *B. japonicus*, mTORC1 regulates digestion of intracellular symbionts according to their nutritional contribution [19]. In *B. azoricus*, by contrast, current evidence is strongest at the tissue level, where immune-associated transcription differs along the anterior–posterior gill axis [13]. These observations should not presently be interpreted as alternative mechanisms. They operate at different biological scales and could prove independent, complementary, or mechanistically linked. Determining whether mTORC1-dependent intracellular regulation occurs within *B. azoricus* bacteriocytes, and how this relates to developmental and spatial heterogeneity across the gill, is therefore one of the most direct tests of the integrated model proposed in Section 7.

The phenoloxidase/prophenoloxidase system represents another conspicuous gap. Phenoloxidase activity has been demonstrated directly in hemocytes of the marine mussel *Mytilus edulis* [77,78], while the broader prophenoloxidase-activating cascade is well established as an important component of invertebrate immunity [79]. However, a comparable system has not, to our knowledge, been characterized in *B. azoricus*. Rather than extrapolating its presence from other invertebrates, determining whether phenoloxidase/prophenoloxidase components occur and function in *B. azoricus* should be considered a specific target for future biochemical and genomic investigation.

Other unresolved questions concern the source and cellular route of symbiont acquisition in newly formed gill tissue, the molecular identity of the soluble hemolymph factors that modify gill responses to different microbial stimuli [13], and whether microbial recognition occurs primarily before or after internalization. Lifelong competence for symbiont acquisition is well established across bathymodioline mussels [38], but newly generated gill tissue is initially aposymbiotic, making it particularly important to distinguish tissue development from active immune permissiveness during subsequent colonization. Together, these questions define experimentally tractable routes for moving from the comparative and correlative framework presently available toward a mechanistic model of immune–symbiosis regulation in *B. azoricus*.

## 10. Conclusions

*Bathymodiolus azoricus* provides an unusually informative system for investigating how innate immunity operates in an animal that maintains dense intracellular chemosynthetic symbionts while remaining exposed to potentially harmful environmental microorganisms. The evidence reviewed here supports neither generalized immune suppression nor a single mechanism of symbiont tolerance. Instead, it indicates regulation distributed across multiple biological scales, from microbial recognition and hemolymph-associated modulation to intracellular symbiont processing, bacteriocyte remodeling, and spatial heterogeneity within the gill.

The strongest species-specific evidence in *B. azoricus* derives from three complementary areas. First, molecular and cellular studies demonstrate an innate-immune repertoire that includes microbial-recognition components, signaling-related transcripts, hemocyte-mediated responses, and antimicrobial-effector candidates [11,23,43]. Second, ex vivo experiments show that gill tissue mounts stimulus-dependent responses to microbial exposure and that soluble hemolymph components modulate these responses [13]. Third, immune-associated gene expression varies along the anterior–posterior gill axis [13]. This spatial pattern is biologically important but not yet fully interpretable: newly formed posterior gill tissue is initially aposymbiotic [38], and lower immune-associated transcription in this region may reflect developmental immaturity, active modulation associated with symbiont acquisition, or both.

Comparative studies across the *Bathymodiolinae* provide additional mechanistic insight but should not be treated as direct evidence for *B. azoricus*. *B. japonicus* demonstrates post-engulfment discrimination between resident symbionts and exogenous bacteria and mTORC1-dependent regulation of symbiont digestion [17,19], while *G./B. platifrons* provides evidence for lineage-specific immune-gene remodeling, functional microbial ligand recognition by GpTLR13, microRNA-associated regulation, developmental variation, and cellular heterogeneity within the gill [18,48,50,62,64,66]. Together, these studies generate testable hypotheses for *B. azoricus* rather than establishing a conserved pathway shared across bathymodioline species.

The host–symbiont–pathogen triad proposed in this review is best understood as an integrative framework rather than a demonstrated signaling pathway. The working hypothesis introduced in Section 1—that this reconciliation is achieved through compartmentalization rather than generalized suppression—remains to be tested directly. Its value lies in bringing together three biological requirements that coexist within the same organ: accommodation and nutritional exploitation of intracellular symbionts, maintenance of antimicrobial competence, and continuous development and remodeling of the gill epithelium. Whether the tissue-level heterogeneity observed in *B. azoricus* and the intracellular checkpoints described in other bathymodiolines represent different manifestations of a shared regulatory principle remains unresolved.

Resolving this question will require cell-resolved and spatial approaches in *B. azoricus*, together with functional analysis of candidate PRRs, hemolymph modulators, nutrient-sensing pathways, antimicrobial effectors, and lysosomal processes. In particular, direct comparison of immune-gene expression between age-matched aposymbiotic and colonized filament regions would provide a first test of the compartmentalization hypothesis proposed in Section 1. Key gaps remain in AMP diversity and function, hemocyte specialization, phenoloxidase/prophenoloxidase biology, the cellular mechanisms of symbiont acquisition, and the relationship between gill development and immune state. Addressing these questions would move *B. azoricus* research beyond comparative and transcriptomic description toward a genuinely mechanistic understanding of how persistent intracellular symbiosis is reconciled with innate immune competence at the deep-sea vent interface.

## Data Availability

No new experimental samples were generated specifically for this review. The sequencing datasets discussed in the manuscript are publicly available through the NCBI Sequence Read Archive. The 2010 *Bathymodiolus azoricus* gill transcriptome is available under SRA accession SRR067874. The independently generated Illumina gill RNA-sequencing dataset is available under BioProject PRJNA1508204, BioSample SAMN62218460, and SRA accession SRR40024054.

## Abbreviations

AIF1	Allograft Inflammatory Factor 1
ALDH	Aldehyde Dehydrogenase
AP-1	Activator Protein 1
CBB	Calvin–Benson–Bassham cycle
LPS	Lipopolysaccharide
MAPK	Mitogen-Activated Protein Kinase
MAMP	Microbe-Associated Molecular Pattern
MeDH	Methanol Dehydrogenase
MMO	Methane Monooxygenase
mTORC1	Mechanistic Target of Rapamycin Complex 1
NCBI	National Center for Biotechnology Information
NF-κB	Nuclear Factor Kappa B
PAMP	Pathogen-Associated Molecular Pattern
PCR	Polymerase Chain Reaction
PGRP	Peptidoglycan Recognition Protein
PO	Phenoloxidase
proPO	Prophenoloxidase
PRR	Pattern-Recognition Receptor
RT-PCR	Reverse Transcription Polymerase Chain Reaction
RTK	Receptor Tyrosine Kinase
SOXB	Sulfur Oxidation Protein/Gene B
SRA	Sequence Read Archive
SRCR	Scavenger Receptor Cysteine-Rich
STAT	Signal Transducer and Activator of Transcription
TFEB	Transcription Factor EB
TLR	Toll-Like Receptor
TLR2	Toll-Like Receptor 2
TLR13	Toll-Like Receptor 13
TNF	Tumor Necrosis Factor
WGA	Wheat Germ Agglutinin

## References

[1] Corliss J.B., Dymond J., Gordon L.I., Edmond J.M., von Herzen R.P., Ballard R.D., Green K., Williams D., Bainbridge A., Crane K. (1979). Submarine thermal springs on the Galápagos Rift. Science.

[2] Spiess F.N., Macdonald K.C., Atwater T., Ballard R., Carranza A., Cordoba D., Cox C., Garcia V.M., Francheteau J., Guerrero J. (1980). East Pacific Rise: Hot springs and geophysical experiments. Science.

[3] Van Dover C.L. (2000). The Ecology of Deep-Sea Hydrothermal Vents.

[4] Childress J.J., Fisher C.R. (1992). The biology of hydrothermal vent animals: Physiology, biochemistry, and autotrophic symbioses. Oceanogr. Mar. Biol. Annu. Rev..

[5] Desbruyères D., Alayse-Danet A.M., Ohta S. (1994). Scientific Parties of biolauand starmerCruises. Deep-sea hydrothermal communities in Southwestern Pacific back-arc basins (the North Fiji and Lau Basins): Composition, microdistribution and food web. Mar. Geol..

[6] Bachraty C., Legendre P., Desbruyères D. (2009). Biogeographic relationships among deep-sea hydrothermal vent faunas at global scale. Deep. Sea Res. Part I Oceanogr. Res. Pap..

[7] Dubilier N., Bergin C., Lott C. (2008). Symbiotic diversity in marine animals: The art of harnessing chemosynthesis. Nat. Rev. Microbiol..

[8] Duperron S., Bergin C., Zielinski F., Blazejak A., Pernthaler A., McKiness Z.P., DeChaine E., Cavanaugh C.M., Dubilier N. (2006). A dual symbiosis shared by two mussel species, *Bathymodiolus azoricus* and *Bathymodiolus puteoserpentis* (Bivalvia: Mytilidae), from hydrothermal vents along the northern Mid-Atlantic Ridge. Environ. Microbiol..

[9] Bordenstein S.R., Theis K.R. (2015). Host biology in light of the microbiome: Ten principles of holobionts and hologenomes. PLoS Biol..

[10] Moran N.A., Sloan D.B. (2015). The hologenome concept: Helpful or hollow?. PLoS Biol..

[11] Bettencourt R., Pinheiro M., Egas C., Gomes P., Afonso M., Shank T., Serrão Santos R. (2010). High-throughput sequencing and analysis of the gill tissue transcriptome from the deep-sea hydrothermal vent mussel *Bathymodiolus azoricus*. BMC Genom..

[12] Bettencourt R., Dando P., Rosa D., Riou V., Colaço A., Sarrazin J., Sarradin P.-M., Serrão Santos R. (2008). Changes of gill and hemocyte-related bio-indicators during long term maintenance of the vent mussel *Bathymodiolus azoricus* held in aquaria at atmospheric pressure. Comp. Biochem. Physiol. Part A Mol. Integr. Physiol..

[13] Bettencourt R., Barros I., Martins E., Martins I., Cerqueira T., Colaço A., Costa V., Rosa D., Froufe H., Egas C., Ray S. (2017). An Insightful Model to Study Innate Immunity and Stress Response in Deep-Sea Vent Animals: Profiling the Mussel *Bathymodiolus azoricus*. Organismal and Molecular Malacology.

[14] Martins E., Figueras A., Novoa B., Serrão Santos R., Moreira R., Bettencourt R. (2014). Comparative study of immune responses in the deep-sea hydrothermal vent mussel *Bathymodiolus azoricus* and the shallow-water mussel *Mytilus galloprovincialis* challenged with *Vibrio* bacteria. Fish Shellfish Immunol..

[15] Barros I., Divya B., Martins I., Vandeperre F., Serrão Santos R., Bettencourt R. (2015). Post-capture immune gene expression studies in the deep-sea hydrothermal vent mussel *Bathymodiolus azoricus* acclimatized to atmospheric pressure. Fish Shellfish Immunol..

[16] Bettencourt R., Costa V., Laranjo M., Rosa D., Pires L., Colaço A., Lopes H., Serrão Santos R. (2011). Out of the deep sea into a land-based aquarium environment: Investigating physiological adaptations in the hydrothermal vent mussel *Bathymodiolus azoricus*. ICES J. Mar. Sci..

[17] Tame A., Maruyama T., Yoshida T. (2022). Phagocytosis of exogenous bacteria by gill epithelial cells in the deep-sea symbiotic mussel *Bathymodiolus japonicus*. R. Soc. Open Sci..

[18] Wang H., He K., Zhang H., Zhang Q., Cao L., Li J., Zhong Z., Chen H., Zhou L., Lian C. (2024). Deciphering deep-sea chemosynthetic symbiosis by single-nucleus RNA-sequencing. eLife.

[19] Tame A., Maruyama T., Ikuta T., Chikaraishi Y., Ogawa N.O., Tsuchiya M., Takishita K., Tsuda M., Hirai M., Takaki Y. (2023). mTORC1 regulates phagosome digestion of symbiotic bacteria for intracellular nutritional symbiosis in a deep-sea mussel. Sci. Adv..

[20] Tame A. (2025). Integrated Regulation of Immunity and Nutritional Symbiosis in Deep-Sea Mussels. Mar. Drugs.

[21] Boutet I., Tanguy A., Le Guen D., Piccino P., Hourdez S., Legendre P., Jollivet D. (2009). Global depression in gene expression as a response to rapid thermal changes in vent mussels. Proc. R. Soc. B Biol. Sci..

[22] Ponnudurai R., Kleiner M., Sayavedra L., Petersen J.M., Moche M., Otto A., Becher D., Takeuchi T., Satoh N., Dubilier N. (2017). Metabolic and physiological interdependencies in the *Bathymodiolus azoricus* symbiosis. ISME J..

[23] Bettencourt R., Dando P., Collins P., Costa V., Allam B., Serrão Santos R. (2009). Innate immunity in the deep sea hydrothermal vent mussel *Bathymodiolus azoricus*. Comp. Biochem. Physiol. Part A Mol. Integr. Physiol..

[24] Allam B., Raftos D. (2015). Immune responses to infectious diseases in bivalves. J. Invertebr. Pathol..

[25] Tame A., Yoshida T., Ohishi K., Maruyama T. (2015). Phagocytic activities of hemocytes from the deep-sea symbiotic mussels *Bathymodiolus japonicus*, *B. platifrons*, and *B. septemdierum*. Fish Shellfish Immunol..

[26] Sekine D., Ohishi K., Nakamura Y., Kusaka C., Tame A., Inoue K., Nakazawa M., Miyake H., Yoshida T., Maruyama T. (2016). Monoclonal antibodies to hemocytes of the deep-sea symbiotic mussel, *Bathymodiolus japonicus*. JAMSTEC Rep. Res. Dev..

[27] Royet J., Gupta D., Dziarski R. (2011). Peptidoglycan recognition proteins: Modulators of the microbiome and inflammation. Nat. Rev. Immunol..

[28] Buriak I., Lanskikh D., Baklanov I., Kozyrev D., Grinchenko A. (2026). C-Type lectins from marine bivalves: Functional diversity and structural insights. Mar. Drugs.

[29] Sarrias M.R., Grønlund J., Padilla O., Madsen J., Holmskov U., Lozano F. (2004). The scavenger receptor cysteine-rich (SRCR) domain: An ancient and highly conserved protein module of the innate immune system. Crit. Rev. Immunol..

[30] Gerdol M., Venier P. (2015). An updated molecular basis for mussel immunity. Fish Shellfish Immunol..

[31] Mitta G., Hubert F., Noël T., Roch P. (1999). Myticin, a novel cysteine-rich antimicrobial peptide isolated from haemocytes and plasma of the mussel *Mytilus galloprovincialis*. Eur. J. Biochem..

[32] Mitta G., Vandenbulcke F., Hubert F., Roch P. (1999). Mussel defensins are synthesised and processed in granulocytes then released into the plasma after bacterial challenge. J. Cell Sci..

[33] Mitta G., Hubert F., Dyrynda E.A., Boudry P., Roch P. (2000). Mytilin B and MGD2, two antimicrobial peptides of marine mussels: Gene structure and expression analysis. Dev. Comp. Immunol..

[34] Rosa R.D., Santini A., Fievet J., Bulet P., Destoumieux-Garzón D., Bachère E. (2011). Big defensins, a diverse family of antimicrobial peptides that follows different patterns of expression in hemocytes of the oyster Crassostrea gigas. PLoS ONE.

[35] Gerdol M., Schmitt P., Venier P., Rocha G., Rosa R.D., Destoumieux-Garzón D. (2020). Functional insights from the evolutionary diversification of big defensins. Front. Immunol..

[36] Détrée C., Chabenat A., Lallier F.H., Satoh N., Shoguchi E., Tanguy A., Mary J. (2016). Multiple I-Type Lysozymes in the Hydrothermal Vent Mussel *Bathymodiolus azoricus* and Their Role in Symbiotic Plasticity. PLoS ONE.

[37] Egas C., Pinheiro M., Gomes P., Barroso C., Bettencourt R. (2012). The transcriptome of *Bathymodiolus azoricus* gill reveals expression of genes from endosymbionts and free-living deep-sea bacteria. Mar. Drugs.

[38] Wentrup C., Wendeberg A., Schimak M., Borowski C., Dubilier N. (2014). Forever competent: Deep-sea bivalves are colonized by their chemosynthetic symbionts throughout their lifetime. Environ. Microbiol..

[39] Bright M., Bulgheresi S. (2010). A complex journey: Transmission of microbial symbionts. Nat. Rev. Microbiol..

[40] Chu H., Mazmanian S.K. (2013). Innate immune recognition of the microbiota promotes host-microbial symbiosis. Nat. Immunol..

[41] Zheng P., Wang M., Li C., Sun X., Wang X., Sun Y., Sun S. (2017). Insights into deep-sea adaptations and host–symbiont interactions: A comparative transcriptome study on *Bathymodiolus* mussels and their coastal relatives. Mol. Ecol..

[42] Saco A., Rey-Campos M., Novoa B., Figueras A. (2020). Transcriptomic response of mussel gills after a *Vibrio splendidus* infection demonstrates their role in the immune response. Front. Immunol..

[43] Détrée C., Lallier F.H., Tanguy A., Mary J. (2017). Identification and gene expression of multiple peptidoglycan recognition proteins (PGRPs) in the deep-sea mussel *Bathymodiolus azoricus*, involvement in symbiosis?. Comp. Biochem. Physiol. Part B Biochem. Mol. Biol..

[44] Mat A.M., Sarrazin J., Markov G.V., Apremont V., Dubreuil C., Eché C., Fabioux C., Klopp C., Sarradin P.-M., Tanguy A. (2020). Biological rhythms in the deep-sea hydrothermal mussel *Bathymodiolus azoricus*. Nat. Commun..

[45] Martins E., Serrão Santos R., Bettencourt R. (2015). Vibrio diabolicus challenge in *Bathymodiolus azoricus* populations from Menez Gwen and Lucky Strike hydrothermal vent sites. Fish Shellfish Immunol..

[46] Barros I., Mendes S., Rosa D., Santos R.S., Bettencourt R. (2016). Vibrio diabolicus immunomodulatory effects on *Bathymodiolus azoricus* during long-term acclimatization at atmospheric pressure. J. Aquac. Res. Dev..

[47] Wang H., Zhang H., Wang M., Chen H., Lian C., Li C. (2019). Comparative transcriptomic analysis illuminates the host-symbiont interactions in the deep-sea mussel *Bathymodiolus platifrons*. Deep Sea Res. Part I Oceanogr. Res. Pap..

[48] Sun J., Zhang Y., Xu T., Zhang Y., Mu H., Zhang Y., Lan Y., Fields C.J., Hui J.H.L., Zhang W. (2017). Adaptation to deep-sea chemosynthetic environments as revealed by mussel genomes. Nat. Ecol. Evol..

[49] Bergstrom K., Xia L. (2022). The barrier and beyond: Roles of intestinal mucus and mucin-type O-glycosylation in resistance and tolerance defense strategies guiding host-microbe symbiosis. Gut Microbes.

[50] Li M., Chen H., Wang M., Zhong Z., Wang H., Zhou L., Zhang H., Li C. (2021). A Toll-like receptor identified in *Gigantidas platifrons* and its potential role in the immune recognition of endosymbiotic methane oxidation bacteria. PeerJ.

[51] Piquet B., Le Panse S., Lallier F.H., Duperron S., Andersen A.C. (2022). “There and back again”—Ultrastructural changes in the gills of Bathymodiolus vent-mussels during symbiont loss: Back to a regular filter-feeding epidermis. Front. Mar. Sci..

[52] Ikuta T., Tame A., Saito M., Aoki Y., Nagai Y., Sugimura M., Inoue K., Fujikura K., Ohishi K., Maruyama T. (2019). Identification of cells expressing two peptidoglycan recognition proteins in the gill of the vent mussel, *Bathymodiolus septemdierum*. Fish Shellfish Immunol..

[53] Wang H., Zhang H., Zhong Z., Sun Y., Wang M., Chen H., Zhou L., Cao L., Lian C., Li C. (2021). Molecular analyses of the gill symbiosis of the bathymodiolin mussel *Gigantidas platifrons*. iScience.

[54] Lee H.-J., Woo Y., Hahn T.-W., Jung Y.M., Jung Y.-J. (2020). Formation and maturation of the phagosome: A key mechanism in innate immunity against intracellular bacterial infection. Microorganisms.

[55] Le Pennec M., Diouris M., Herry A. (1988). Endocytosis and lysis of bacteria in gill epithelium of *Bathymodiolus thermophilus*, *Thyasira flexuosa* and *Lucinella divaricata* (Bivalvia, Mollusca). J. Shellfish Res..

[56] Rabanal-Ruiz Y., Korolchuk V.I. (2018). mTORC1 and nutrient homeostasis: The central role of the lysosome. Int. J. Mol. Sci..

[57] Ballabio A., Bonifacino J.S. (2020). Lysosomes as dynamic regulators of cell and organismal homeostasis. Nat. Rev. Mol. Cell Biol..

[58] Yu J., Wang M., Liu B., Yue X., Li C. (2019). Gill symbionts of the cold-seep mussel *Bathymodiolus platifrons*: Composition, environmental dependency and immune control. Fish Shellfish Immunol..

[59] Settembre C., Di Malta C., Polito V.A., Garcia Arencibia M., Vetrini F., Erdin S., Uckac Erdin S., Huynh T., Medina D., Colella P. (2011). TFEB links autophagy to lysosomal biogenesis. Science.

[60] Vogeler S., Carboni S., Li X., Joyce A. (2021). Phylogenetic analysis of the caspase family in bivalves: Implications for programmed cell death, immune response and development. BMC Genom..

[61] Kádár E., Bettencourt R., Costa V., Serrão Santos R., Lobo-da-Cunha A., Dando P.R. (2005). Experimentally induced endosymbiont loss and re-acquirement in the hydrothermal vent bivalve *Bathymodiolus azoricus*. J. Exp. Mar. Biol. Ecol..

[62] Li M., Chen H., Wang M., Zhong Z., Zhou L., Li C. (2020). Identification and characterization of endosymbiosis-related immune genes in deep-sea mussels *Gigantidas platifrons*. J. Oceanol. Limnol..

[63] Wong Y.H., Sun J., He L.S., Chen L.G., Qiu J.-W., Qian P.-Y. (2015). High-throughput transcriptome sequencing of the cold seep mussel *Bathymodiolus platifrons*. Sci. Rep..

[64] Huang J., Huang P., Lu J., Wu N., Lin G., Zhang X., Cao H., Geng W., Zhai B., Xu C. (2022). Gene expression profiles provide insights into the survival strategies in deep-sea mussel (*Bathymodiolus platifrons*) of different developmental stages. BMC Genom..

[65] Lin G., Wen B., Huang J., Liang X., Xu C., Chen Y., Wu N., Lu J. (2025). Comparative transcriptome analysis revealed host defense responses in endosymbiotic gill of *Bathymodiolus* mussels inhabiting cold seeps and hydrothermal vents. J. Oceanol. Limnol..

[66] Chen H., Wang M., Zhang H., Wang H., Zhou L., Zhong Z., Cao L., Lian C., Sun Y., Li C. (2021). microRNAs facilitate comprehensive responses of *Bathymodiolinae* mussel against symbiotic and nonsymbiotic bacteria stimulation. Fish Shellfish Immunol..

[67] Lorion J., Kiel S., Faure B., Kawato M., Ho S.Y.W., Marshall B., Tsuchida S., Miyazaki J.-I., Fujiwara Y. (2013). Adaptive radiation of chemosymbiotic deep-sea mussels. Proc. R. Soc. B.

[68] Li Y., Tassia M.G., Waits D.S., Bogantes V.E., David K.T., Halanych K.M. (2019). Genomic adaptations to chemosymbiosis in the deep-sea seep-dwelling tubeworm *Lamellibrachia luymesi*. BMC Biol..

[69] Ikuta T., Igawa K., Tame A., Kuroiwa T., Kuroiwa H., Aoki Y., Takaki Y., Nagai Y., Ozawa G., Yamamoto M. (2016). Surfing the vegetal pole in a small population: Extracellular vertical transmission of an ‘intracellular’ deep-sea clam symbiont. R. Soc. Open Sci..

[70] Ozawa G., Shimamura S., Takaki Y., Takishita K., Ikuta T., Barry J.P., Maruyama T., Fujikura K., Yoshida T. (2017). Ancient occasional host switching of maternally transmitted bacterial symbionts of chemosynthetic vesicomyid clams. Genome Biol. Evol..

[71] McFall-Ngai M., Nyholm S.V., Castillo M.G. (2010). The role of the immune system in the initiation and persistence of the *Euprymna scolopes*–*Vibrio fischeri* symbiosis. Semin. Immunol..

[72] Nyholm S.V., Graf J. (2012). Knowing your friends: Invertebrate innate immunity fosters beneficial bacterial symbioses. Nat. Rev. Microbiol..

[73] Huang B., Zhang L., Xu F., Tang X., Li L., Wang W., Liu M., Zhang G. (2019). Oyster versatile IKKα/βs are involved in Toll-like receptor and RIG-I-like receptor signaling for innate immune response. Front. Immunol..

[74] Kawai T., Akira S. (2010). The role of pattern-recognition receptors in innate immunity: Update on Toll-like receptors. Nat. Immunol..

[75] McFall-Ngai M., Hadfield M.G., Bosch T.C.G., Carey H.V., Domazet-Lošo T., Douglas A.E., Dubilier N., Eberl G., Fukami T., Gilbert S.F. (2013). Animals in a bacterial world, a new imperative for the life sciences. Proc. Natl. Acad. Sci. USA.

[76] Foo J.L., Ling H., Lee Y.S., Chang M.W. (2017). Microbiome engineering: Current applications and its future. Biotechnol. J..

[77] Coles J.A., Pipe R.K. (1994). Phenoloxidase activity in the haemolymph and haemocytes of the marine mussel Mytilus edulis. Fish Shellfish Immunol..

[78] Luna-Acosta A., Breitwieser M., Renault T., Thomas-Guyon H. (2017). Recent findings on phenoloxidases in bivalves. Mar. Pollut. Bull..

[79] Cerenius L., Söderhäll K. (2004). The prophenoloxidase-activating system in invertebrates. Immunol. Rev..

